# Early‐life anthropometry and colorectal cancer risk in adulthood: Global Cancer Update Programme (CUP Global) systematic literature review and meta‐analysis of prospective studies

**DOI:** 10.1002/ijc.35461

**Published:** 2025-05-28

**Authors:** Moniek van Zutphen, Auke J. C. F. Verkaar, Fränzel J. B. van Duijnhoven, Trudy Voortman, Monica L. Baskin, Rajiv Chowdhury, Ellen Copson, Sarah J. Lewis, Lynette Hill, John Krebs, Matty P. Weijenberg, Jacob C. Seidell, Yikyung Park, Jennifer L. Baker, Mojgan Amiri, Tosca O. E. de Crom, Erand Llanaj, Amber Meulenbeld, Macarena Lara, Yuchan Mou, Vanessa L. Z. Gordon‐Dseagu, Esther M. González‐Gil, Georgios Markozannes, Konstantinos K. Tsilidis, Doris S. M. Chan, Ellen Kampman, Dieuwertje E. Kok

**Affiliations:** ^1^ Division of Human Nutrition and Health Wageningen University and Research Wageningen The Netherlands; ^2^ Department of Epidemiology, Erasmus MC University Medical Center Rotterdam Rotterdam The Netherlands; ^3^ UPMC Hillman Cancer Center Pittsburgh Pennsylvania USA; ^4^ Department of Global Health Florida International University Miami Florida USA; ^5^ Cancer Sciences Academic Unit University of Southampton Southampton UK; ^6^ Department of Population Health Sciences, Bristol Medical School University of Bristol Bristol UK; ^7^ World Cancer Research Fund International London UK; ^8^ Department of Zoology University of Oxford Oxford UK; ^9^ Department of Epidemiology Maastricht University Maastricht The Netherlands; ^10^ Faculty of Science, Department of Health Sciences Vrije Universiteit Amsterdam Amsterdam The Netherlands; ^11^ Department of Surgery Washington University in St. Louis St. Louis Missouri USA; ^12^ Center for Clinical Research and Prevention Copenhagen University Hospital – Bispebjerg and Frederiksberg Copenhagen Denmark; ^13^ Nutrition and Metabolism Branch, International Agency for Research on Cancer World Health Organization Lyon France; ^14^ Department of Epidemiology and Biostatistics, School of Public Health Imperial College London London UK; ^15^ Department of Hygiene and Epidemiology University of Ioannina Medical School Ioannina Greece

**Keywords:** anthropometry, birthweight, colorectal cancer risk, early‐life exposure, systematic review

## Abstract

While adult anthropometric measures are well‐studied in relation to colorectal cancer (CRC) risk, the impact of early‐life anthropometry remains unclear. We conducted a systematic literature review and meta‐analysis examining early‐life anthropometry, including birth size, height and adiposity and adult CRC risk. We searched Medline, Embase, Web of Science and CENTRAL. Early‐life stages were categorised as at birth, infancy (0 to <2 years), childhood (2 to 9 years), adolescence (10 to 19 years) and young adulthood (18 to 25 years). Random‐effects meta‐analyses were conducted when ≥3 prospective observational studies provided sufficient information; otherwise, results were descriptively synthesised. We included 37 publications, and evidence was graded by the Global Cancer Update Programme Expert Panel. Higher birthweight (relative risk [RR] per 1000 g: 1.09, 95% confidence interval [CI] 1.01–1.16; 8 studies, 8134 cases) and young adult body mass index (BMI, RR per 5 kg/m^2^: 1.12, 95% CI 1.07–1.17; 16 studies, 20,365 cases) were associated with higher CRC risk. Associations for young adult BMI were most pronounced for colon cancer (RR per 5 kg/m^2^: 1.15, 95% CI: 1.06–1.24). Descriptive synthesis showed that childhood and adolescent BMI were also associated with higher colon and/or CRC risk. Evidence for all the above associations was graded by the Expert Panel as “strong‐probable.” Additionally, there was “limited‐suggestive” evidence linking higher birthweight to higher colon cancer risk, taller childhood height to higher CRC risk, early‐life adiposity—measured by BMI pictograms—to higher colon and CRC risk and higher young adult BMI to rectal cancer risk. Other exposure‐outcome associations were graded as “limited‐no conclusion.” Altogether, these results imply that larger body size during early life is associated with higher adult CRC risk.

## Graphical abstract

What's new?

While adult anthropometry is linked to colorectal cancer (CRC) risk, associations between anthropometric measures across early‐life stages and CRC risk remain unclear. In this study, the authors systematically reviewed and meta‐analysed early‐life anthropometry and adult CRC risk across early‐life stages. Higher birthweight as well as higher BMI in childhood, adolescence, and young adulthood were associated with an increased CRC risk. This evidence was graded as strong‐probable. The findings imply that larger body size in early‐life is relevant to CRC risk, which could help inform lifestyle recommendations for CRC prevention.

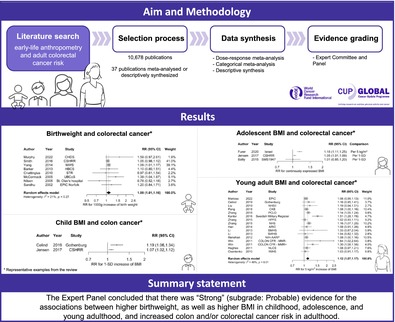

**Abbreviations**
BMI: body mass index
CI: confidence interval
CRC: colorectal cancer
CUP Global: Global Cancer Update Programme
MR: Mendelian randomisation
NOS: Newcastle Ottawa Quality Assessment Scale
RR: relative risk
SD: standard deviation
WCRF: World Cancer Research Fund

## 1 INTRODUCTION

Colorectal cancer (CRC) is the third most common cancer worldwide.^1^ While adiposity and adult attained height are associated with a higher risk of CRC,^2^, ^3^ most research focused on adult exposures. Early‐life anthropometry, including birth size, height and adiposity, may also affect cancer risk later in life as early life is a critical developmental period,^4^ lifelong habits are often established early in life^5^ and exposure to early‐life risk factors can lead to prolonged cumulative exposure.^4^

Studies have shown that adiposity during early life is associated with CRC risk in adulthood.^6^, ^7^, ^8^, ^9^, ^10^ However, associations between anthropometry and CRC risk may differ between early‐life stages, that is, at birth, infancy, childhood, adolescence and young adulthood. To date, only one review including studies from the Copenhagen School Health Records Register has provided results by specific early‐life stages.^11^ They reported that body mass index (BMI) and height during childhood and adolescence, as well as higher birthweight, were associated with the risk of several cancers, including colon cancer.^11^ No other reviews have examined associations between anthropometry in different early‐life stages and CRC risk in adulthood, except for birthweight. High birthweight has been associated with a higher CRC risk compared with low birthweight,^11^, ^12^ although there was no conclusive evidence in linear and nonlinear dose–response meta‐analyses.^12^, ^13^

Given the lack of consistent evidence, we conducted a systematic literature review and dose–response meta‐analyses to summarise the evidence on birth size and various anthropometric measures during infancy, childhood, adolescence and young adulthood in relation to CRC risk through the Global Cancer Update Programme (CUP Global)—the flagship research programme of the WCRF network. The CUP Global Expert Panel used pre‐defined criteria to grade the evidence.

## 2 METHODS

We report this review according to the Preferred Reporting Items for Systematic Reviews and Meta‐Analyses (Table S1).

### 2.1 Search strategy

We conducted a systematic search for relevant publications in Medline, Embase, Web of Science and CENTRAL from inception up to 7 November 2022. Detailed search queries are provided in Text S1. Additionally, we reviewed the CUP Global database and reference lists of relevant reviews and meta‐analyses.

### 2.2 Study selection

This review included prospective studies—cohort, case‐cohort, nested case–control, intervention and Mendelian randomisation (MR) studies—while excluding case–control studies, cross‐sectional studies and case reports. Eligible studies had to provide relative risk (RR; e.g., hazard ratio, odds ratio) estimates and 95% confidence intervals (CIs) for the association between early‐life anthropometric factors and colorectal, colon, or rectal cancer risk in adulthood. We defined early‐life as age ≤ 25 years, encompassing birth, infancy (0 to <2 years), childhood (2 to 9 years), adolescence (10 to 19 years) and young adulthood (18 to 25 years). The age range for adolescence and young adulthood slightly overlapped because for 18‐ to 19‐year‐olds BMI could have been reported either as BMI *z*‐scores (appropriate for adolescents) or as BMI values (appropriate for [young] adults) in the respective papers. All studies that examined adolescent exposures also included individuals aged <17 years at exposure assessment. Study selection was conducted in duplicate by multiple independently working authors, with disagreements resolved through consensus. To avoid duplication of results from the same study populations within an early‐life stage, we prioritised publications with the largest sample size. Publications with repeatedly measured exposures could be included in multiple life stages, but within each early‐life stage, only one exposure measurement was retained (at the age most reported across studies). Studies were excluded if the study population was not exclusively aged ≤25 years, if the outcome focused solely on cancer mortality, or if the publication was not written in English. Additionally, we excluded studies focusing solely on early‐onset CRC, since early‐onset CRC might differ from later‐onset CRC in terms of aetiology and risk factors.^14^

### 2.3 Data extraction

Study and participants' characteristics, along with results for each exposure‐outcome association, were extracted into a pilot‐tested data extraction sheet. When the studies reported multiple RRs and 95% CIs for different CRC subtypes, adjustment models, or subgroups, we extracted all relevant estimates. Extracted data are shown in Tables S2–S6. At least 10% of the extracted data was randomly checked for accuracy by a second author and was fully accurate.

### 2.4 Risk of bias assessment

Risk of bias for all studies, except MR studies, was assessed using the Newcastle Ottawa Quality Assessment Scale (NOS).^15^ The NOS evaluates selection of the study population, comparability of groups and ascertainment of the outcome (Tables S2–S6). To ensure accuracy, at least 90% of publications were independently assessed by two authors. Discrepancies were resolved through consensus. The NOS score ranges from 0 to 9.^15^ We considered scores ≥7 as well‐designed studies with low risk of bias.^16^

### 2.5 Statistical analyses

For linear and nonlinear dose–response meta‐analyses, we included publications reporting at least three exposure categories with known mean or median values, along with RR, 95% CI and number of cases and person‐years (or number of people) per category. When exposure category values, cases, or person‐years were missing, we performed standard imputations.^17^, ^18^ The midpoint of each exposure category was used for corresponding RR estimates, and the width of open‐ended extreme categories was assumed to be the same as the adjacent interval. Studies unable to undergo imputation due to missing information (on exposure category values or on both cases and person‐years) were excluded from the review (*n* = 1). Publications reporting RR and 95% CI for linear trends, but not for exposure categories, were only included in linear dose–response meta‐analyses.

Linear and nonlinear dose–response meta‐analyses were conducted separately for anthropometric measures within each early‐life stage and their association with colorectal, colon (or its subsites), or rectal cancer risk. Linear and non‐linear dose–response meta‐analyses were conducted when at least three or five (non‐MR) studies provided data, respectively, consistent with previous meta‐analyses conducted as part of CUP Global.^19^, ^20^ Otherwise, results were descriptively synthesised. For this review, we selected RRs and 95% CIs from the most adjusted models that excluded adjustment for the exposure of interest in adult life (e.g., adult BMI or adult height), when available.

For linear dose–response meta‐analyses, we utilised RRs and 95% CIs reported for the linear trend in original publications or estimated the linear trend based on category‐specific results.^21^, ^22^, ^23^ For young adult BMI, we excluded the underweight category (BMI <18.5 kg/m^2^ or as defined by studies) when the linear trend was estimated. Linear trends for childhood and adolescent BMI were reported directly and did not require estimation. For BMI pictograms (range 1–9), we reported RRs for overweight (pictogram 5–9) versus non‐overweight (pictogram 1–4); when studies reported alternative comparisons, we used the Hamling method to estimate relevant RRs.^24^ When only subgroup‐specific RR estimates were available, these were pooled in a fixed‐effect model to derive an overall study estimate before summarising with the other studies using the DerSimonian–Laird random‐effects model.^25^ For birthweight, additional categorical meta‐analyses were performed comparing either high or low birthweight to normal birthweight (2500–4000 g) as five out of eight identified studies reported results for these clinically relevant categories. Between‐study heterogeneity was assessed by Cochran's *Q* test and *I* ^2^,^26^ with thresholds of 30% and 50% categorising low, moderate and high percentage of inconsistency across studies attributed to heterogeneity rather than chance. Egger's test was used to assess small study effects as an indication of publication bias when more than 10 studies were available, with pooled publications counted as single studies.^27^

One‐stage nonlinear dose–response meta‐analyses were conducted using restricted cubic splines to explore shapes of the associations.^21^ Three a priori chosen knots were placed at the 10th, 50th and 90th percentiles of the exposure distribution. Based on the number of available studies (≥5), these analyses were possible for birthweight (reference 3000 g) and young adult BMI (reference 18.5 kg/m^2^).

To assess consistency of results, we conducted subgroup analyses when at least two strata in at least three studies were available. Subgroup analyses were conducted for birthweight/BMI assessment method, sex and geographic location. Influence of single studies on the summary RR estimate was examined by leave‐one‐out analyses, where studies were omitted one‐by‐one from the meta‐analyses. Leave‐one‐out analyses were only performed when at least three studies remained in the analyses. Statistical significance was set at a two‐sided alpha level of 0.05, except for Egger's test where we used 0.1. All statistical analyses were conducted using R (version 4.3.3) with the ‘meta’ (version 7.0.0)^28^ and ‘dosresmeta’ (version 2.0.1)^21^ packages.

### 2.6 Evidence grading criteria

Preliminary judgements were made by the CUP Global Expert Committee on Cancer Incidence. These judgements, alongside the literature review and supporting evidence, were used by the CUP Global Expert Panel to grade the evidence into strong (subgrades: convincing, probable, or substantial effect on risk unlikely) or limited (subgrades: limited‐suggestive or limited‐no‐conclusion) based on pre‐defined grading criteria to assess quantity, consistency, magnitude and precision of the summary estimates, existence of a dose–response, study design, risk of bias, generalisability and mechanistic plausibility of the results (for overview of criteria, see Table S7). Forest plots were visually inspected for consistency of associations, noting the direction and size of the RR estimates and overlapping of the CIs across included studies as part of evidence grading. Neither the CUP Global Expert Committee on Cancer Incidence nor the CUP Global Expert Panel was involved in the review and analyses of the literature.

## 3 RESULTS

Figure S1 outlines the study selection process. We identified 54 relevant publications on early‐life anthropometry and colorectal, colon, or rectal cancer risk. After excluding 17 publications^29^, ^30^, ^31^, ^32^, ^33^, ^34^, ^35^, ^36^, ^37^, ^38^, ^39^, ^40^, ^41^, ^42^, ^43^, ^44^, ^45^ not meeting inclusion criteria (Table S8), 37 publications were included.^12^, ^13^, ^46^, ^47^, ^48^, ^49^, ^50^, ^51^, ^52^, ^53^, ^54^, ^55^, ^56^, ^57^, ^58^, ^59^, ^60^, ^61^, ^62^, ^63^, ^64^, ^65^, ^66^, ^67^, ^68^, ^69^, ^70^, ^71^, ^72^, ^73^, ^74^, ^75^, ^76^, ^77^, ^78^, ^79^, ^80^ Of these, 9 non‐MR publications focused on birth size,^13^, ^46^, ^47^, ^48^, ^49^, ^50^, ^51^, ^52^, ^53^ 6 on children,^54^, ^55^, ^56^, ^57^, ^58^, ^59^ 5 on adolescents,^56^, ^57^, ^60^, ^61^, ^62^ 15 on young adults^54^, ^57^, ^63^, ^64^, ^65^, ^66^, ^67^, ^68^, ^69^, ^70^, ^71^, ^72^, ^73^, ^74^, ^75^ and 2 on changes across early‐life stages,^54^, ^56^ with some studies investigating more than one early‐life stage. No studies were identified for infants. Seven publications were MR studies^12^, ^63^, ^76^, ^77^, ^78^, ^79^, ^80^

Among the publications on conventional observational studies, 16 were from Europe,^13^, ^47^, ^48^, ^49^, ^50^, ^51^, ^52^, ^54^, ^55^, ^56^, ^58^, ^59^, ^61^, ^63^, ^67^, ^72^ 10 from the United States,^46^, ^53^, ^57^, ^62^, ^64^, ^65^, ^68^, ^70^, ^73^, ^75^ 3 from Asia,^60^, ^66^, ^69^ 1 from Australia^74^ and 1 was multinational.^71^ MR studies only included people from European ancestry.^12^, ^63^, ^76^, ^77^, ^78^, ^79^, ^80^ The majority of studies (*n* = 27; 79%) had a NOS quality score of ≥7^46^, ^47^, ^48^, ^49^, ^50^, ^51^, ^53^, ^54^, ^55^, ^56^, ^57^, ^58^, ^59^, ^60^, ^61^, ^63^, ^64^, ^66^, ^67^, ^68^, ^69^, ^70^, ^72^, ^74^, ^75^ and were therefore considered to be of high quality, whereas three studies had a score of 6,^65^, ^69^, ^71^ three studies had a score of 5^13^, ^62^, ^71^ and one study had a score of 4.^52^

### 3.1 Birth size

**FIGURE 1:**
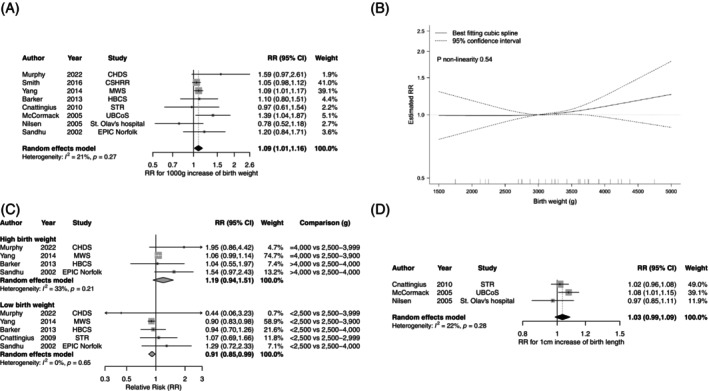
Associations between birthweight, birth length and colorectal cancer risk in adulthood. This figure shows the results from (A) linear dose–response association between birthweight and colorectal cancer risk; (B) nonlinear dose–response association between birthweight and colorectal cancer risk (*p*_nonlinearity_ = .54); (C) comparison of high and low birthweight versus normal birthweight and colorectal cancer risk; (D) linear dose–response association between birth length and colorectal cancer risk. The diamond represents the summary relative risk (RR) estimate, with its width indicating the 95% confidence interval (CI). The squares and horizontal lines show study‐specific RRs and their 95% CIs. The area of each square reflects the study's weight in the meta‐analysis. The increment unit in the linear dose–response analyses is 1000 g or 1 cm.

For birthweight, the linear meta‐analysis included 8134 CRC cases from eight studies.^13^, ^46^, ^47^, ^48^, ^49^, ^50^, ^51^, ^52^ Each 1000 g increase in birthweight was associated with a 9% increase in CRC risk (summary RR = 1.09, 95% CI 1.01–1.16), showing low heterogeneity (*I* ^2^ = 21%, *p*_heterogeneity_ = .27) (Figure 1A). There was no evidence for nonlinearity (*p*‐nonlinearity = .54)^13^, ^46^, ^48^, ^49^, ^50^, ^52^ (Figure 1B). Descriptive syntheses by tumour subsites included 2 studies with a total of 2090 colon cancer cases^47^, ^53^ and 1 study with 961 rectal cancer cases.^47^ The studies examining colon cancer indicated that each 1000 g increase in birthweight was associated with a statistically significant 14%–19% higher risk (data not shown).^47^, ^53^ The study examining rectal cancer suggested a potential nonlinear association, with an inverse association for birthweights above 3500 g.^47^

Additional meta‐analyses comparing low (<2500 g) with normal birthweight (2500–4000 g)^13^, ^46^, ^48^, ^49^, ^52^ or comparing high (≥4000 g) with normal birthweight^13^, ^46^, ^48^, ^52^ included 4830 and 4750 CRC cases, respectively. Results aligned with linear meta‐analyses, although the positive association between high birthweight and CRC risk was not statistically significant (summary RR = 1.19, 95% CI 0.94–1.51, *I* ^2^ = 33%, *p*_heterogeneity_ = .21) (Figure 1C).

For birth length, a total of 754 CRC cases from three studies were included in the linear meta‐analysis.^49^, ^50^, ^51^ There was no clear association between each 1‐cm increase and CRC risk (summary RR = 1.03, 95% CI 0.99–1.09, *I* ^2^ = 22%, *p*_heterogeneity_ = .28) (Figure 1D).

MR studies did not support the positive association between birthweight and CRC risk (Table S9). The four available MR studies showed no clear associations between genetically predicted birthweight and CRC risk, with RRs ranging from 0.69 to 1.22, none of which reached statistical significance.^12^, ^76^, ^77^, ^80^ No MR studies were identified for birth length.

### 3.2 Child anthropometry

**FIGURE 2:**
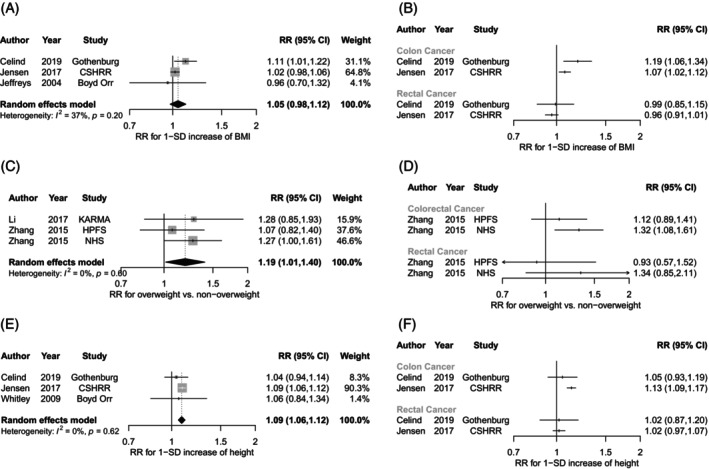
Associations between child anthropometry and colorectal, colon, or rectal cancer risk in adulthood. This figure shows the results from (A) linear dose–response associations between body mass index (BMI) *z*‐scores and colorectal cancer risk; (B) descriptive synthesis of linear dose–response associations between BMI *z*‐scores and colon and rectal cancer risk; (C) comparison of overweight (BMI pictogram ≥5) versus non‐overweight (BMI pictogram <5) and colon cancer risk; (D) descriptive synthesis of comparison of overweight (BMI pictogram ≥5) versus non‐overweight (BMI pictogram <5) and colorectal and rectal cancer risk; (E) linear dose–response associations between height *z*‐scores and colorectal cancer risk; (F) descriptive synthesis of linear dose–response associations between height z‐scores and colon or rectal cancer risk. The black diamond represents the summary relative risk (RR) estimate, with its width indicating the 95% confidence interval (CI). The squares and horizontal lines show study‐specific RRs and their 95% CIs. The area of each grey square reflects the study's weight in the meta‐analysis. The increment unit in (A), (B), (E), and (F) is 1 standard deviation (SD).

For BMI during childhood, the linear meta‐analysis included 4494 CRC cases from 3 studies.^54^, ^56^, ^59^ There was no clear association between each 1 standard deviation (SD) increase in child BMI and CRC risk (summary RR = 1.05, 95% CI 0.98–1.12, *I* ^2^ = 37%, *p*_heterogeneity_ = .20) (Figure 2A). Descriptive synthesis by tumour subsite included 2 studies reporting stratified results for colon (2744 cases) and rectal (1712 cases) cancer separately, indicating a higher risk for colon cancer, but not for rectal cancer^54^, ^56^ (Figure 2B).

For child BMI pictograms, the meta‐analysis included 1884 colon cancer cases from 3 studies.^55^, ^57^ Being overweight was associated with a 19% higher colon cancer risk compared with not being overweight (summary RR = 1.19, 95% CI 1.01–1.40, *I* ^2^ = 0%, *p*_heterogeneity_ = .60) (Figure 2C). Additional descriptive synthesis included 2 studies that reported on CRC (2100 cases) and rectal cancer (451 cases) risk.^57^ Results showed that a positive association may be present for CRC (RRs 1.12 and 1.32, only 1 statistically significant), but was not shown for rectal cancer (Figure 2D).

For child height, the linear meta‐analysis included 4515 CRC cases from 3 studies.^54^, ^56^, ^58^ Each 1‐SD increase was associated with a 9% increased CRC risk (summary RR = 1.09, 95% CI 1.06–1.12, *I* ^2^ = 0%, *p*_heterogeneity_ = .62) (Figure 2E). Descriptive synthesis by tumour subsite, including two studies, showed no clear associations between child height and risk of colon or rectal cancer^54^, ^56^ (Figure 2F).

MR studies seemed to support the positive association between childhood BMI and CRC risk^76^, ^77^, ^78^ (Table S9). The three available MR studies reported RRs >1 for genetically predicted childhood BMI or obesity and CRC risk, although only one study reached statistical significance.^76^ No MR studies were identified for child height.

### 3.3 Adolescent anthropometry

**FIGURE 3:**
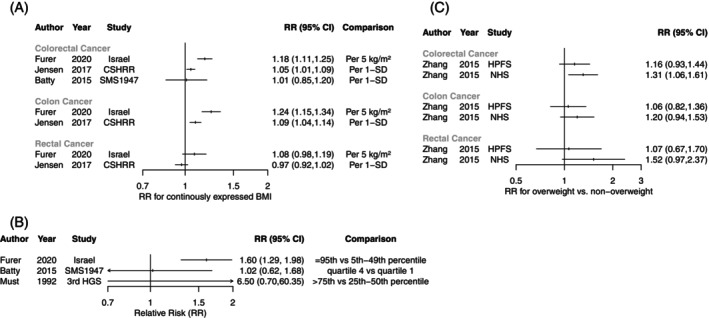
Descriptive synthesis of association between adolescent adiposity and colorectal, colon, and rectal cancer risk in adulthood. This figure shows the results for (A) linear dose–response association between body mass index (BMI) and colorectal, colon, and rectal cancer risk; (B) comparison of high versus low BMI and colorectal cancer risk; (C) comparison of overweight (BMI pictogram ≥5) versus non‐overweight (BMI pictogram <5) and colorectal, colon, or rectal cancer risk. The squares and horizontal lines show study‐specific relative risks (RRs) and their 95% confidence intervals (CIs).

For BMI during adolescence, the descriptive synthesis included 5643 CRC cases from 4 studies with different comparisons.^56^, ^60^, ^61^, ^62^ The 2 largest studies (5511 cases combined) indicated that higher BMI was associated with increased CRC risk (RRs 1.05 per 1‐SD and 1.18 per 5 kg/m^2^)^56^, ^60^ (Figure 3A). These larger studies also reported higher risk for colon, but not rectal, cancer. Also, studies reporting on BMI categories showed RRs >1 (Figure 3B). The largest study (2801 CRC cases) reported that obesity (≥95th BMI percentile) was associated with increased CRC risk compared with the lower healthy weight range (5th to 49th BMI percentile) (RR = 1.60, 95% CI 1.29–1.98),^60^ but the 2 smaller studies (132 cases combined) showed non‐significant associations.^61^, ^62^

For adolescent BMI pictograms, the descriptive synthesis included 2100 CRC cases from 2 cohorts^57^ (Figure 3C). Consistent with the results for adolescent BMI, overweight adolescents had an increased CRC risk compared with their non‐overweight peers (RRs 1.16 and 1.31, only the latter being statistically significant), with the association being most pronounced in women. When stratified by tumour subsite, RRs >1 were observed for colon and rectal cancer risk, though none were statistically significant.

For adolescent height, the descriptive synthesis included 4040 CRC cases from 1 study.^56^ Each 1‐SD increase in height was associated with a 9% increase in CRC risk (RR = 1.09, 95% CI 1.05–1.13).

MR studies supported the positive association between adolescent adiposity and CRC risk (Table S9). The two available MR studies reported RRs >1 for genetically predicted adiposity at age 10 and CRC risk, although associations did not reach statistical significance.^63^, ^79^ One MR study, stratified by tumour subsite, also reported RRs >1 for colon and rectal cancer risk.^79^ No MR studies were identified for adolescent height.

### 3.4 Young adult anthropometry

**FIGURE 4:**
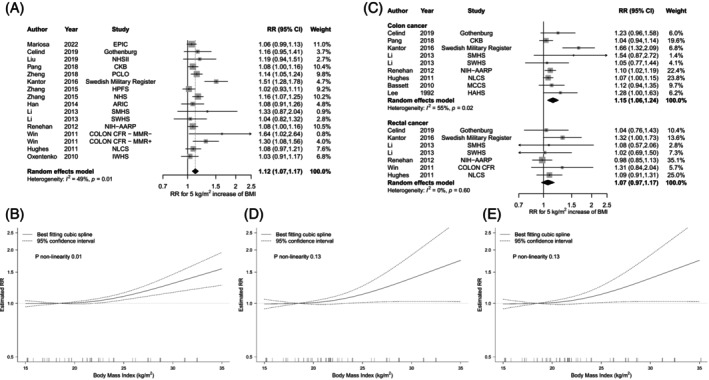
Linear and nonlinear dose–response associations between young adult body mass index (BMI) and colorectal, colon, and rectal cancer risk in adulthood. This figure shows the results from (A) linear dose–response associations between BMI and colorectal cancer risk; (B) nonlinear dose–response association between BMI and colorectal cancer risk (*p*_nonlinearity_ = .01); (C) linear dose–response association between BMI and colon or rectal cancer risk; (D) nonlinear dose–response association between BMI and colon cancer risk (*p*_nonlinearity_ = .13); (E) nonlinear dose–response association between BMI and rectal cancer risk (*p*_nonlinearity_ = .72). The black diamond represents the summary relative risk (RR) estimate, with its width indicating the 95% confidence interval (CI). The squares and horizontal lines show study‐specific RRs and their 95% CIs. The area of each grey square reflects the study's weight in the meta‐analysis. The increment unit is 5 kg/m^2^.

For young adult BMI, the linear meta‐analysis included 20,365 CRC cases from 16 studies.^54^, ^57^, ^63^, ^64^, ^65^, ^66^, ^67^, ^68^, ^69^, ^70^, ^71^, ^72^, ^73^ Each 5 kg/m^2^ increase in BMI was associated with a 12% increased CRC risk (summary RR = 1.12, 95% CI 1.07–1.17) with moderate heterogeneity (*I* ^2^ = 49%, *p*_heterogeneity_ = .01) (Figure 4A). Despite some heterogeneity in the magnitude of RRs among the studies, all consistently reported RRs >1. In the nonlinear meta‐analysis,^57^, ^64^, ^65^, ^66^, ^67^, ^68^, ^69^, ^70^, ^71^, ^72^, ^73^ there was evidence of nonlinearity, suggesting a somewhat steeper risk increase at higher BMI, but no higher risk at underweight BMI (*p*‐nonlinearity = .01; Figure 4B). Egger's test indicated small study effects such as publication bias (*p* = .06). Publication bias was explained by Kantor et al.^67^ (Figure S2). After omitting this study, the positive association with CRC risk remained (summary RR = 1.09, 95% CI 1.06–1.13; *I* ^2^ = 10%).

When stratified by tumour subsite, each 5 kg/m^2^ increase in young adult BMI was associated with a higher risk of colon cancer (summary RR = 1.15, 95% CI 1.06–1.24, *I* ^2^ = 55%, *p*_heterogeneity_ = .02) (Figure 4C), proximal colon cancer (summary RR = 1.07, 95% CI 1.01–1.14, *I* ^2^ = 0%, *p*_heterogeneity_ = .53) (Figure S3A) and distal colon cancer (summary RR = 1.10, 95% CI: 1.00–1.21, *I* ^2^ = 41%, *p*_heterogeneity_ = .18) (Figure S3B). For rectal cancer risk, there might also be a positive association, but this did not reach statistical significance (summary RR = 1.07, 95% CI 0.97–1.17; *I* ^2^ = 0%, *p*_heterogeneity_ = .60) (Figure 4C). There was no evidence of nonlinearity for colon^67^, ^69^, ^70^, ^72^, ^74^, ^75^ and rectal^67^, ^69^, ^70^, ^72^ cancer risk (Figure 4D,E).

No MR studies were identified for young adult adiposity.

### 3.5 Changes in body mass index during early life

One study reported that an above‐average increase in BMI (versus average increase) between ages 7 and 13 years was associated with a higher risk of colon, but not rectal cancer.^56^ The other study indicated that BMI changes between 8 and 20 years were not associated with either colon or rectal cancer risk.^54^

### 3.6 Consistency of findings

For birthweight, we were able to stratify our meta‐analyses by birthweight ascertainment method; while for young adult BMI, we stratified by sex, geographical location and BMI ascertainment method (Table 1). Summary RRs were generally consistent across the subgroups, except for the association with colon cancer which was stronger in studies in which young adult BMI was objectively measured (summary RR = 1.38, 95% CI 1.15–1.67, *I* ^2^ = 45%) compared with recalled during adult life (summary RR = 1.08, 95% CI 1.03–1.13, *I* ^2^ = 0%; *p*_subgroup_ = .01). Results for young adults remained stable in leave‐one‐out analysis. Birthweight results were influenced by one study^13^; the associations of both the dose–response and low versus normal analyses were no longer statistically significant, while the association for high versus normal birthweight reached statistical significance when excluding the study by Yang et al. (Figure S4).

**TABLE 1:** Subgroup meta‐analyses of birthweight and young adult body mass index (BMI) in relation to colorectal, colon and rectal cancer risk in adulthood by sex, geographic location and exposure assessment method.

Subgroup	*N* studies, cases	RR (95% CI)	*I* ^2^	*p*_subgroup‐heterogeneity_	Included publications
**Birthweight, per 1000 g**					
Colorectal cancer					
Weight assessment				0.63	
Birth record	5 studies 1097 cases	1.14 (0.90–1.43)	45%		^46^, ^48^, ^49^, ^50^, ^51^
Recalled	3 studies, 7037 cases	1.07 (1.01–1.13)	0%		^13^, ^47^, ^52^
**Young adult BMI, per 5 kg/m** ^**2**^					
Colorectal cancer					
Sex				0.19	
Men	10 studies, >8852 cases	1.15 (1.07–1.24)	60%		^54^, ^57^, ^63^, ^65^, ^67^, ^68^, ^70^, ^71^, ^72^, ^73^
Women	10 studies, >8289 cases	1.09 (1.04–1.14)	0%		^57^, ^63^, ^64^, ^65^, ^68^, ^69^, ^70^, ^71^, ^72^, ^73^
Geographic location				0.11	
China	3 studies, 3297 cases	1.08 (1.01–1.16)	0%		^66^, ^69^
Europe	4 studies, 6665 cases	1.17 (1.02–1.17)	80%		^54^, ^63^, ^67^, ^72^
Multinational	2 studies, 658 cases	1.34 (1.13–1.59)	0%		^71^
United States	7 studies, 9945 cases	1.09 (1.05–1.14)	16%		^57^, ^64^, ^65^, ^68^, ^70^, ^73^
Colon cancer					
Sex				0.20	
Men	7 studies, 3438 cases	1.25 (1.10–1.42)	53%		^54^, ^67^, ^69^, ^70^, ^72^, ^74^, ^75^
Women	4 studies, 2188 cases	1.19 (1.10–1.28)	0%		^69^, ^70^, ^72^, ^74^
Geographic location				0.48	
Australia	1 study, 569 cases	1.12 (0.94–1.35)	—		^74^
China	3 studies, 2186 cases	1.05 (0.96–1.15)	0%		^66^, ^69^
Europe	3 studies, 2298 cases	1.28 (0.98–1.68)	85%		^54^, ^67^, ^72^
United States	2 studies, 2318 cases	1.13 (1.01–1.26)	19%		^26^, ^31^
BMI assessment				0.01	
Measured	3 studies, 1024 cases	1.38 (1.15–1.67)	45%		^54^, ^67^, ^75^
Recalled	6 studies, 6347 cases	1.08 (1.03–1.13)	0%		^66^, ^69^, ^70^, ^72^, ^74^
Rectal cancer					
Sex				0.40	
Men	5 studies, 1592 cases	1.08 (0.97–1.21)	0%		^10^, ^23^, ^25^, ^26^, ^28^
Women	3 studies, 671 cases	0.99 (0.83–1.18)	0%		^69^, ^70^, ^72^

*Note*: The results are organized by exposure (bold) and outcome (underlined).

Abbreviations: CI, confidence interval; RR, relative risk.

### 3.7 Evidence grading

Overall, the evidence from prospective observational studies led to 8 associations being classified as ‘strong’ (subgrade: probable) (Table 2). The evidence showed higher risk of colon and/or CRC with higher birthweight and with higher BMI during childhood, adolescence and young adulthood. Lower CRC risk was shown with low versus normal birthweight.

**TABLE 2:** Evidence grades and main findings from the meta‐analyses and descriptive synthesis of early‐life anthropometry and colorectal cancer risk in adulthood.

Evidence grade		Anthropometry in early‐life and risk of colorectal cancer in adulthood			
		Exposure	Outcome	Summary of findings RR (95% CI)	Conclusions
Strong evidence	Convincing	—	—	—	
	Probable	**Decreases risk**			
		Low birthweight	Colorectal cancer	RR low (<2500 g) vs. normal (2500–4000 g) = 0.91(95% CI 0.85–0.99), *I* ^2^ 0%,5 studies, 4830 cases	The evidence showed lower risk with a low birthweight versus normal birthweight. No heterogeneity.
		**Increases risk**			
		Birthweight	Colorectal cancer	RR per 1000 g = 1.09 (95% CI 1.01–1.16), *I* ^2^ 21%, 8 studies, 8134 cases	The evidence showed higher risk with higher birthweight. Low heterogeneity.
		Childhood BMI	Colon cancer	BMI per 1‐SD increase: 2 studies, 2744 cases, no meta‐analysis RRs ranged from 1.07 to 1.19, both 95% CIs did not include 1	The evidence showed a higher risk with higher BMI. Evidence based on 2 large cohort studies which both showed significant results. Supportive evidence from MR studies.
		Adolescent BMI	Colorectal cancer	4 studies, 5643 cases, no meta‐analysis. RRs of 2 largest studies (5511 cases): 1.05 per 1‐SD and 1.18 per 5 kg/m^2^ for BMI as continuous exposure, both 95% CIs did not include 1	
			Colon cancer	BMI per 1‐SD increase: 2 studies, 4378 cases, no meta‐analysis RRs ranged from 1.09 to 1.24, both 95% CIs did not include 1	
		Young adult BMI	Colorectal cancer	RR per 5 kg/m^2^ = 1.12 (95% CI 1.07–1.17), I^2^ 49%,16 studies, 20,365 cases, *p*_Egger_ .06	The evidence showed a higher risk with higher BMI. Moderate heterogeneity existed, but all RRs >1.
			Colon cancer	RR per 5 kg/m^2^ = 1.15 (95% CI 1.06–1.24), *I* ^2^ 55%, 9 studies, 7371 cases	
			Proximal colon cancer	RR per 5 kg/m^2^ = 1.07 (95% CI 1.01–1.14), *I* ^2^ 0% 6 studies, 5411 cases	The evidence showed a higher risk with higher BMI. No heterogeneity.
Limited evidence	Limited suggestive	**Increases risk**			
		Birthweight	Colon cancer	Birthweight per 1000 g: 2 studies, 2090 cases, no meta‐analysis. RRs ranged from 1.14 to 1.19, none of the 95% CIs included 1	The evidence tended to show higher risk with higher birthweight. Evidence was based on 2 cohort studies.
		Childhood height	Colorectal cancer	RR per 1 cm = 1.09 (95% CI 1.06–1.12), *I* ^2^ 0%, 3 studies, 4515 cases	The evidence tended to show higher risk with being taller. Only the largest study reached statistical significance.
		Childhood adiposity	Colon cancer	RR overweight vs. non‐overweight (based on BMI pictograms) = 1.19 (95% CI 1.01–1.40), *I* ^2^ 0%, 3 studies, 1884 cases	The evidence tended to show higher risk with being overweight versus non‐overweight. While the summary RR was significant, only 1 study was significant.
			Colorectal cancer	Overweight vson‐overweight (based on BMI pictograms): 2 studies, 2100 cases, no meta‐analysis. RRs ranged from 1.12 to 1.32, 1 95% CI did not include 1	The evidence tended to show higher risk with being overweight versus non‐overweight. Evidence based on 2 large well‐designed cohort studies (only 1 statistically significant). Supportive evidence from MR studies. A limitation is that each study included either men or women.
		Adolescent adiposity	Colorectal cancer	Overweight vs. non‐overweight (based on BMI pictograms): 2 studies, 2100 cases, no meta‐analysis. RRs ranged from 1.16 to 1.31, 1 95% CI did not include 1	
		Young adult BMI	Distal colon cancer	RR per 5 kg/m^2^ = 1.10 (95% CI 1.00–1.21), *I* ^2^ 41%, 3 studies, 2772 cases	The evidence tended to show higher risk with higher BMI. The pooled estimate was borderline significant with moderate heterogeneity.
			Rectal cancer	RR per 5 kg/m^2^ = 1.07 (95% CI 0.97–1.17), *I* ^2^ 0%, 7 studies, 2144 cases	The evidence tended to show higher risk with higher BMI, but the pooled estimate did not reach statistical significance. No heterogeneity.
	Limited—no conclusion	Birthweight	Rectal cancer	1 study, 961 cases, no meta‐analysis. RR 4.5 kg versus 3.5 kg = 0.77 (95% CI 0.61–0.96), nonlinear association	Inconsistent findings, not all results statistically significant.
		High birthweight	Colorectal cancer	RR high (>4000 g) vs. normal (2500–4000 g) = 1.19 (95% CI 0.94–1.51), *I* ^2^ 33% 4 studies, 4750 cases	
		Birth length	Colorectal cancer	RR per 1 cm = 3 studies, 754 cases 1.03 (95% CI 0.99–1.09), *I* ^2^ 22%	
		Childhood height	Colon cancer	Height per 1‐SD: 2 studies, 2744 cases, no meta‐analysis. RRs ranged from 1.05 to 1.13, 1 95% CI did not include 1	
			Rectal cancer	Height per 1‐SD: 2 studies, 1712 cases, no meta‐analysis. Both RRs 1.02, both 95% CIs included 1	
		Childhood BMI	Colorectal cancer	RR per 1‐SD increase = 1.05 (95% CI 0.98–1.12), *I* ^2^ 37%, 3 studies, 4494 cases	
			Rectal cancer	BMI per 1‐SD increase: 2 studies, 1712 cases, no meta‐analysis. RRs ranged from 0.96 to 0.99, all 95% CIs included 1	
		Childhood adiposity	Rectal cancer	Overweight vs. non‐overweight (based on BMI pictograms): 2 studies, 451 cases, no meta‐analysis. RRs ranged from 0.93 to 1.34, all 95% CIs included 1	
		Adolescent height	Colorectal cancer	Height per 1‐SD increase: 1 study, 4040 cases, no meta‐analysis. RR 1.09, 95% CI did not include 1	
		Adolescent adiposity	Colon cancer	Overweight vs. non‐overweight (based on BMI pictograms): 2 studies, 1649 cases, no meta‐analysis. RRs ranged from 1.06 to 1.20, both 95% CIs included 1	
			Rectal cancer	Overweight vs. non‐overweight (based on BMI pictograms): 2 studies, 451 cases, no meta‐analysis RRs ranged from 1.07 to 1.52, both 95% CIs included 1 BMI: 2 studies, 2686 cases, no meta‐analysis. RRs 0.97 per 1‐SD and 1.08 per 5 kg/m^2^, both 95% CIs included 1	
		Change in BMI during early life	Colorectal/colon/rectal cancer		Limited number of studies available (*n* = 2)

Abbreviations: BMI, body mass index; CI, confidence interval; RR, relative risk; SD, standard deviation.

Seven other associations were classified as ‘limited‐suggestive’ based on the predetermined grading criteria. The evidence tended to link higher birthweight with higher colon cancer risk, taller childhood height with higher CRC risk, early‐life adiposity (measured by BMI pictograms) with higher colorectal and colon cancer risk and higher young adult BMI with higher rectal cancer risk.

All other associations were classified as ‘limited‐no conclusion’. While associations with ‘limited‐no conclusion’ evidence grades concerning colon cancer or CRC risk generally pointed in the same direction as the other associations receiving a stronger evidence grade, they were based on few studies. For rectal cancer, all associations, except for young adult BMI, were graded as ‘limited‐no conclusion’, due to the availability of only one or two studies.

## 4 DISCUSSION

We systematically reviewed findings from prospective observational studies on the association between early‐life anthropometrics and CRC risk in adulthood. Early‐life anthropometry, particularly higher birthweight and BMI during childhood, adolescence and young adulthood, was associated with a higher risk of colon cancer and CRC. Findings for childhood and adolescent adiposity were generally supported by MR studies.

The positive association between birthweight and CRC risk was graded as ‘strong‐probable’ by the CUP Global Expert Panel. Higher birthweight was associated with higher CRC risk and low versus normal birthweight with lower CRC risk, with low or no between‐study heterogeneity. MR studies did not show a clear association between genetically predicted birthweight and CRC risk. However, three out of five MR studies, including the largest MR study to date^81^ which was published after our search, found a suggestive positive association of birthweight with CRC risk.^76^, ^80^, ^81^ Birthweight reflects a complex interplay of genetic, nutritional and other environmental factors affecting foetal growth. Factors resulting in higher birthweight, or its consequences, may impact CRC development through largely uncharacterised biological mechanisms. One potential mechanism linking birthweight to CRC is epigenetic programming. Epigenome‐wide association studies demonstrated that DNA methylation profiles in neonatal blood were associated with birthweight.^82^ Likewise, various studies imply a role for aberrant gene‐specific DNA methylation patterns in blood in CRC risk.^83^, ^84^, ^85^

The positive associations between early‐life BMI and colorectal and/or colon cancer risk were graded as ‘strong‐probable’. Sixteen studies consistently showed that higher BMI in young adults was associated with higher CRC risk, mirroring the well‐established associations between adult BMI and CRC risk.^3^ In contrast, fewer studies were available for BMI during adolescence and childhood. Our descriptive synthesis indicated that higher BMI in childhood was associated with higher risk of colon cancer, while higher BMI during adolescence was associated with risk of both colon cancer and CRC. Although these findings were based on two observational studies within each early‐life stage, these studies were large, well‐designed and showed narrow confidence intervals. Findings were further supported by MR studies, leading the Expert Panel to grade this evidence as ‘strong‐probable’. In addition, higher adiposity during childhood and adolescence, measured by BMI pictograms, also tended to be associated with higher CRC risk whereas higher young adult BMI tended to be associated with higher rectal cancer risk. This body of evidence was graded as ‘limited‐suggestive’. Being taller during childhood also tended to be associated with higher CRC risk, reflecting associations observed in adults,^3^ however findings were mainly based on one large study.^56^ Mechanisms underlying associations between early‐life adiposity and CRC risk remain poorly understood but may involve inflammation and immune cell dysfunction.^86^ Children with overweight or obesity show higher circulating levels of proinflammatory markers like C‐reactive protein, interleukine‐6 and tumor necrosis factor‐α compared with children with normal weight, pointing towards potential chronic low‐grade inflammation.^87^, ^88^ This inflammatory state may encourage tumour growth, potentially through impairments in natural killer cell functioning.^86^ Initial studies in children have suggested that natural killer cells from children with obesity are activated, metabolically stressed and functionally impaired, potentially weakening antitumor immunity long before cancer develops.^86^

Several limitations should be considered when interpreting the results of our systematic literature review and meta‐analyses. While we observed positive associations between early‐life adiposity and CRC risk in adulthood, it remains unclear whether these associations are independent of later‐life exposures. This is mainly because individuals with obesity in childhood often still have obesity in adulthood due to behavioural, environmental and genetic factors.^89^, ^90^ Although observational and MR studies have both reported positive associations for early‐life adiposity and CRC risk, one MR study suggested that adult adiposity may explain the observed association.^79^ Specifically, the association between genetically predicted early‐life BMI and CRC risk was attenuated to the null after including genetically predicted adult BMI in the model.^79^ The only formal mediation analyses examining whether adult BMI mediates the association between early‐life anthropometry and CRC risk focused on birthweight.^91^ They reported a positive association between birthweight and CRC risk, with adult BMI partially mediating this association.^91^ Our review focused exclusively on early‐life periods and did not examine BMI trajectories extending beyond young adulthood. While trajectory modelling could provide further insights by tracking body growth patterns across life stages, such studies are scarce,^65^, ^89^, ^92^, ^93^ often begin in young adulthood,^65^, ^92^ with few participants having overweight or obesity in early life^65^, ^89^, ^92^ limiting the ability to assess whether normalising BMI reduces CRC risk. In summary, it remains unclear whether early life is a sensitive period for CRC risk or if prolonged exposure to high adiposity is the contributing factor.

A further limitation is that many included studies relied on recall data rather than objectively measured anthropometrics, likely affecting accuracy of exposure assessment. Our subgroup analyses, however, revealed that exposure ascertainment method for birthweight did not alter conclusions. Furthermore, many studies failed to adjust for potential confounders or were potentially over‐adjusted for adult lifestyle factors. Only three studies took premature birth into account and no studies adjusted for early‐life physical activity. Nevertheless, five young adult studies that did not adjust for adult lifestyle factors showed similar results to those that did. Also MR studies, which are less prone to confounding, supported positive associations between early‐life BMI and CRC risk. In addition, although our subgroup analysis showed that results did not differ between China, Europe and the United States, most studies were conducted in high‐income countries which may limit generalisability to low‐ and middle‐income countries, were the double burden of malnutrition is present.^94^ Finally, some analyses may have had limited power due to the small number of available studies. Based on predetermined grading criteria—including the number of studies and precision of summary estimates—several associations received ‘limited‐no conclusion’ grades. However, these associations generally aligned with associations of comparable exposure with strong evidence grades, suggesting similar trends despite limited power.

Despite its limitations, this is the most comprehensive systematic review and meta‐analysis on early‐life anthropometry and CRC risk conducted to date. Evidence was systematically synthesised and graded by the CUP Global Expert Panel following standardised grading criteria. This review uniquely addressed exposures across different early‐life stages, including birth‐related characteristics, childhood, adolescence and young adulthood. Inclusion of both conventional observational studies and MR studies adds robustness, as each approach has distinct strengths and limitations. Future studies can expand the evidence base, particularly in under‐researched areas like infancy, childhood and adolescence.

## 5 CONCLUSIONS

There is ‘strong’ (subgrade: probable) evidence for the associations between higher birthweight and early‐life BMI and a higher risk of colon cancer and/or CRC. There is ‘limited‐suggestive’ evidence for the positive associations between birthweight and colon cancer, early‐life adiposity during childhood and adolescence and colon cancer and/or CRC, young adult BMI and rectal cancer, and being taller during childhood and CRC risk. Other exposure‐outcome associations received a ‘limited‐no conclusion’ grading.

This study highlights the potential to inform lifestyle recommendations aimed at reducing CRC risk. Although birthweight and height are not direct targets for cancer prevention, their associations with CRC provide insights into the possible early origins of the disease. Further research with repeated measurements during early life may indicate the potential for how reducing adiposity in early life might impact adult CRC risk.^94^ Further studies are also needed in low‐ and middle‐income countries, where the double burden of malnutrition is prevalent.^94^

## AUTHOR CONTRIBUTIONS

**Moniek van Zutphen:** Writing – original draft; writing – review and editing; visualization; formal analysis; investigation; methodology; data curation. **Auke J. C. F. Verkaar:** Writing – original draft; writing – review and editing; formal analysis; methodology; investigation; data curation. **Fränzel J. B. van Duijnhoven:** Supervision; writing – review and editing. **Trudy Voortman:** Conceptualization; methodology; supervision; writing – review and editing; funding acquisition. **Monica L. Baskin:** Writing – review and editing. **Rajiv Chowdhury:** Writing – review and editing. **Ellen Copson:** Writing – review and editing. **Sarah J. Lewis:** Writing – review and editing. **Lynette Hill:** Writing – review and editing. **John Krebs:** Writing – review and editing. **Matty P. Weijenberg:** Writing – review and editing. **Jacob C. Seidell:** Writing – review and editing. **Yikyung Park:** Writing – review and editing. **Jennifer L. Baker:** Writing – review and editing. **Mojgan Amiri:** Investigation; writing – review and editing. **Tosca O. E. de Crom:** Investigation; writing – review and editing. **Erand Llanaj:** Investigation; writing – review and editing. **Amber Meulenbeld:** Investigation; writing – review and editing. **Macarena Lara:** Writing – review and editing; data curation; investigation. **Yuchan Mou:** Investigation; writing – review and editing. **Vanessa L. Z. Gordon‐Dseagu:** Project administration; writing – review and editing. **Esther M. González‐Gil:** Writing – review and editing. **Georgios Markozannes:** Supervision; writing – review and editing; methodology. **Konstantinos K. Tsilidis:** Writing – review and editing. **Doris S. M. Chan:** Supervision; writing – review and editing; methodology. **Ellen Kampman:** Funding acquisition; supervision; conceptualization; writing – review and editing. **Dieuwertje E. Kok:** Investigation; supervision; writing – review and editing.

## FUNDING INFORMATION

This work was funded by the World Cancer Research Fund network of charities: American Institute for Cancer Research (AICR), World Cancer Research Fund (WCRF), Wereld Kanker Onderzoek Fonds (WKOF) (Project reference number: CUP_2021_001). The process was based on the method developed by WCRF International's Methodology Task Force for the WCRF/AICR Second Expert Report.

## CONFLICT OF INTEREST STATEMENT

Jennifer Lynn Baker declares no conflicts of interest relevant to this manuscript and has received consulting fees from Novo Nordisk A/S. Ellen Copson declares no conflicts of interest relevant to this manuscript and has received research support from SECA. All other authors declare no conflicts of interest.

## ETHICS STATEMENT

This review is part of a larger review project on diet, physical activity, and anthropometry in early life and CRC risk for which the protocol was registered at PROSPERO (CRD42020213415).

## Supporting information


**Data S1.** Supporting Information.

## Data Availability

Only publicly available data were used in this study. Data sources and handling of these data are described in the methods section. Further details are available from the corresponding author upon request.
