# Supplementary material for: Early‐life anthropometry and colorectal cancer risk in adulthood: Global Cancer Update Programme (CUP Global) systematic literature review and meta‐analysis of prospective studies
Source: Int J Cancer. 2025 May 28;157(6):1094–109. doi: 10.1002/ijc.35461 (PMC12280858; doi:10.1002/ijc.35461)
Supplement: Supplementary file 1 — Data S1. Supporting Information. [file IJC-157-1094-s001.pdf]

<u>**Supplementary Material**</u>

**Early-life anthropometry and colorectal cancer risk in adulthood: Global Cancer Update Programme (CUP Global) systematic literature review and meta-analysis**

<u>Authors</u>

Moniek van Zutphen, Auke J.C.F. Verkaar, Fränzel J.B. van Duijnhoven, Trudy Voortman, Monica L. Baskin, Rajiv Chowdhury, Ellen Copson, Sarah J. Lewis, Lynette Hill, John Krebs, Matty P. Weijenberg, Jacob C. Seidell, Yikyung Park, Jennifer L. Baker, Mojgan Amiri, Tosca O.E. de Crom, Erand Llanaj, Amber Meulenbeld, Macarena Lara, Yuchan Mou, Vanessa L.Z. Gordon-Dseagu, Esther M. González-Gil, Georgios Markozannes, Konstantinos K. Tsilidis, Doris S.M. Chan, Ellen Kampman, Dieuwertje E. Kok

# Table of Contents

Supplementary Text S1 Search strategy and search terms per database ........ 3

Supplementary Text S1A Search terms used for Embase.com ........ 3

Supplementary Text S1B Search terms used for Medline (OVID) ........ 4

Supplementary Text S1C Search terms used for Cochrane CENTRAL register of Trials ........ 6

Supplementary Text S1D Search terms used for Web of Science Core Collection ........ 7

Supplementary Table S1 PRISMA 2020 Checklist ........ 9

Supplementary Table S2 Results from studies examining the association between birth size and risk of colorectal cancer in adulthood ........ 13

Supplementary Table S3 Results from studies examining the association between childhood body fatness and height and risk of colorectal cancer in adulthood ........ 17

Supplementary Table S4 Results from studies examining the association between adolescent body fatness and height and risk of colorectal cancer in adulthood ........ 19

Supplementary Table S5 Results from studies examining the association between young adult BMI and risk of colorectal cancer in adulthood ........ 21

Supplementary Table S6 Results from studies examining the association between change in early life BMI and risk of colorectal cancer in adulthood ........ 25

Supplementary Table S7 Grading criteria for evidence on diet, nutrition, physical activity and cancer incidence ........ 26

Supplementary Table S8. Studies excluded and reason of exclusion from the potentially eligible studies after further inspection ........ 27

Supplementary Table S9 Results from Mendelian randomization (MR) studies examining the association between genetically predicted anthropometry in early life and risk of colorectal cancer in adulthood ........ 28

Supplementary Figure S1: Flowchart of study selection process ........ 30

Supplementary Figure S2: Funnel plot of studies included in the linear dose-response meta-analysis of young adult BMI and colorectal cancer risk in adulthood ............................................ 31
Supplementary Figure S3: Meta-analyses young adult adiposity stratified by tumour subsites ..... 32
Supplementary Figure S4: Leave-one-out analyses of birthweight ................................................ 33
References ........................................................................................................................ 34

**Supplementary Text S1 Search strategy and search terms per database**

The search was constructed and performed by a medical information search specialist from Erasmus University Medical Library, Rotterdam, The Netherlands. The search strategy used the following elements: colorectal cancer; diet/nutrition, physical activity, and/or body size; and time of exposure during early life.

## Supplementary Text S1A Search terms used for Embase.com

('large intestine tumor'/exp OR 'colorectal polyp'/de OR 'colon polyp'/exp OR 'rectum polyp'/de OR (((cancer* OR neoplas* OR tumor* OR tumour* OR polyp OR polyps OR carcino* OR adenocarcinoma* OR adenoma* OR sarcoma* OR malign*) NEAR/6 (large-intestin* OR colon* OR rectum OR rectal OR colorect* OR desmoid* OR sigmoid* OR gut)) OR ((mass) NEAR/6 (large-intestin* OR colon* OR rectum OR rectal OR colorectal* OR desmoid* OR sygmoid* OR gut) NOT body-mass) OR ((cancer* OR neoplas* OR tumor* OR polyp OR polyps OR mass OR carcino* OR adenocarcinoma* OR adenoma* OR sarcoma* OR malign*) NEAR/3 (bowel*) NOT small-bowel*) OR CRC):Ab,ti,kw) AND (child/exp OR infant/exp OR adolescent/exp OR adolescence/exp OR 'child behavior'/de OR 'childhood obesity'/de OR 'adolescent obesity'/de OR 'young adult'/de OR pediatrics/exp OR childhood/exp OR 'child nutrition'/de OR 'infant nutrition'/exp OR 'child development'/de OR 'child growth'/de OR 'prematurity'/de OR Pregnancy/exp OR 'pregnant woman'/de OR 'mother'/de OR 'prenatal period'/exp OR 'newborn period'/exp OR (child* OR infan* OR adolescen* OR preadolescen* OR ((young OR early) NEXT/1 (adult* OR women OR men)) OR prenatal* OR maternal* OR baby OR babies OR newborn* OR (new NEXT/1 born*) OR neonat* OR prematur* OR pre-matur* OR kid OR kids OR toddler* OR teen* OR boy* OR girl* OR minors OR underag* OR (under NEXT/1 (age*)) OR juvenil* OR youth* OR kindergar* OR puber* OR pubescen* OR prepubescen* OR prepubert* OR pediatric* OR paediatric* OR schoolchild* OR highschool* OR primary-school* OR preterm* OR pregnan* OR postnatal* OR post-natal* OR Preschool* OR Pre-school* OR Perinatal* OR Perinatal* OR Sucking OR prenatal* OR antenatal*):ab,ti,kw) AND ('nutrition'/de OR food/exp OR 'food intake'/exp OR diet/exp OR 'physical activity'/de OR 'body weight'/exp OR 'body weight disorder'/exp OR 'supplementation'/exp OR 'dietary intake'/exp OR 'body composition'/exp OR 'muscle mass'/exp OR 'food frequency questionnaire'/de OR 'food diary'/de OR '24 hour dietary recall'/de OR 'carbohydrate'/de OR 'mineral'/de OR 'vitamin'/exp OR 'nutritional deficiency'/exp OR 'grain'/exp OR 'calorie'/exp OR 'meat'/exp OR nut/exp OR vegetable/exp OR 'dairy product'/exp OR 'fruit'/exp OR 'egg'/exp OR 'sugar'/exp OR starch/de OR 'fatty acid'/exp OR 'lactation'/exp OR 'anthropometric parameters'/exp OR 'skinfold thickness'/exp OR exercise/de OR 'sport'/exp OR 'physical inactivity'/de OR housekeeping/de OR recreation/exp OR 'computer addiction'/exp OR 'standing'/de OR 'sitting'/de OR 'feeding behavior'/exp OR 'nutritional assessment'/de OR 'probiotic agent'/exp OR 'prebiotic agent'/de OR 'leisure'/de OR 'fitness'/de OR 'physical performance'/de OR 'body constitution'/de OR 'weight, mass and size'/exp OR 'skinfold thickness'/de OR 'dual energy X ray absorptiometry'/de OR 'Diet therapy'/de OR Protein/exp OR 'soft drink'/de OR 'sweetened beverage'/exp OR 'gluten'/de OR 'caffeine'/de OR 'fruit pulp'/de OR 'polysaccharide'/de OR 'sweetening agent'/de OR 'hydrogenated oil'/de OR 'retinol'/de OR 'carotenoid'/de OR 'pyridoxine'/de OR 'cobalamin'/de OR 'iodine'/de OR 'phytoestrogen'/de OR (nutrition* OR food* OR feeding OR eating OR diet* OR dietary OR supplement OR supplements OR ((physical* OR leisure* OR outdoor*) NEAR/3 (activ* OR inactiv*)) OR fitness OR

(Physical* NEAR/3 (exertion* OR endurance* OR condition* OR Performan* OR education*)) OR Play-time OR chores OR (Planned NEAR/3 (exercise)) OR ((skinfold* OR skin-fold*) NEAR/3 (thickness OR parameter*)) OR dexa OR bioimpedence OR (Formula NEAR/3 (fed OR feading OR baby)) OR soft-drink* OR softdrink* OR ((sweet* OR sugar*) NEAR/3 beverage*) OR total-fat OR trans-fat OR gluten OR Fibre* OR Wholegrain* OR wheat* OR Wholewheat* OR soy OR soybean* OR soya OR bread OR milk OR caffein* OR fruit-pulp* OR juice* OR polysaccharid* OR ((fluid* OR water) NEAR/3 (intake* OR consum*)) OR drinking OR drink OR drinks OR linol*-acid* OR Folic-acid* OR sweetening OR sweetener* OR hydrogenated-oil* OR retinol* OR carotenoid* OR Niacin OR pyridoxin* OR cobalamin* OR iodine OR phytoestrogen* OR ((hip OR head OR chest) NEAR/3 circumfer*) OR bio-impedence OR Adiposity OR (body NEAR/3 (weight OR size OR mass OR height OR composition* OR fat OR fatness OR distribut* OR water OR potassium* OR muscle* OR Length)) OR pufa OR mufa OR ((fat OR energy OR iron OR fatty-acid* OR amino-acid* OR protein* OR calcium OR selenium OR magnesium OR potassium OR zinc OR copper OR phosphorus OR salt OR lipid* OR fiber* OR Carbohydrate* OR fish OR mineral* OR vitamin* OR calor* OR meat OR vegetable* OR alcohol OR beverage* OR starch OR grain* OR polyphenol* OR nut OR nuts OR fruit OR dairy OR egg OR eggs OR sugar OR sodium) NEAR/3 (consumption OR underconsumption OR overconsumption OR intake OR deficien* OR restrict*)) OR hunger OR sedentary* OR (energ* NEAR/3 (expenditure* OR balance* OR density)) OR (muscle NEAR/3 (mass* OR weight*)) OR bmi OR (weight NEAR/3 (loss OR gain OR reduction*)) OR ((resistance OR strength OR functional) NEAR/3 (training)) OR tai-chi OR yoga OR walking OR cycling OR bike-riding OR sports OR sport OR folinic-acid* OR folate OR breastfe* OR breast-fe* OR obes* OR overweight* OR (birth NEXT/1 (size OR weight)) OR (small* NEAR/3 (gestation*)) OR birthweight* OR breast-milk OR human-milk OR phytochemical* OR phyto-chemical* OR lactat* OR anthropomet* OR (waist NEAR/3 (hip or circumferen*)) OR ((skinfold OR skin-fold) NEAR/3 thickness) OR (weight NEAR/3 (loss OR change* OR gain OR reduction*)) OR birth-weight OR birthweight OR exercise OR housekeeping* OR house-keeping* OR recreation* OR video-game* OR videogame* OR (activit* NEAR/3 (Household OR Occupation* OR aerobic OR Cardiovascular OR Endurance OR Life-style OR Lifestyle OR Free-living OR Transportation*)) OR (Transportation* NEAR/3 (mode)) OR (standing NOT long-standing) OR sitting OR (Television NEAR/3 (viewing OR watching OR time)) OR (screen NEAR/3 time) OR probiotic* OR prebiotic* OR pro-biotic* OR pre-biotic* OR ascorbic-acid* OR cholecalciferol* OR riboflavin* OR tocopherol* OR thiamin*):ab,ti,kw) NOT ('survivor'/mj OR 'cancer survivor'/mj OR (survivor* OR cancer-treat* OR cancer-therap*):ti) NOT [conference abstract]/lim NOT ([animals]/lim NOT [humans]/lim)

Supplementary Text S1B Search terms used for Medline (OVID)

(large intestine tumor/ OR colorectal polyp/ OR colon polyp/ OR rectum polyp/ OR (((cancer* OR neoplas* OR tumor* OR tumour* OR polyp OR polyps OR carcino* OR adenocarcinoma* OR adenoma* OR sarcoma* OR malign*) ADJ6 (large-intestin* OR colon* OR rectum OR rectal OR colorect* OR desmoid* OR sigmoid* OR gut)) OR ((mass) ADJ6 (large-intestin* OR colon* OR rectum OR rectal OR colorectal* OR desmoid* OR sygmoid* OR gut) NOT body-mass) OR ((cancer* OR neoplas* OR tumor* OR polyp OR polyps OR mass OR carcino* OR adenocarcinoma* OR adenoma* OR sarcoma* OR malign*) ADJ3 (bowel*) NOT small-bowel*) OR CRC).ab,ti,kf.) AND (exp Child/ OR exp Infant/ OR exp Adolescent/ OR exp "Child Behavior"/ OR exp "Parent Child Relations"/ OR exp "Pediatrics"/ OR "Child Nutrition Sciences"/ OR "Infant nutritional physiological phenomena"/ OR exp

"Child Welfare"/ OR "Child Development"/ OR exp "Child Health Services"/ OR exp "Child Care"/ OR "Child Rearing"/ OR exp "Child development Disorders, Pervasive"/ OR "Child Psychiatry"/ OR "Child Psychology"/ OR "Hospitals, Pediatric"/ OR exp "Intensive Care Units, Pediatric"/ OR exp Pregnancy / OR Pregnant Women / OR Mothers / OR (child* OR infan* OR adolescen* OR preadolescen* OR ((young OR early) ADJ (adult* OR women OR men)) OR prenatal* OR maternal* OR baby OR babies OR newborn* OR (new ADJ born*) OR neonat* OR prematur* OR pre-matur* OR kid OR kids OR toddler* OR teen* OR boy* OR girl* OR minors OR underag* OR (under ADJ (age*)) OR juvenil* OR youth* OR kindergar* OR puber* OR pubescen* OR prepubescen* OR prepubert* OR pediatric* OR paediatric* OR schoolchild* OR highschool* OR primary-school* OR preterm* OR pregnan* OR postnatal* OR post-natal* OR Preschool* OR Pre-school* OR Perinatal* OR Perinatal* OR Sucking OR prenatal* OR antenatal*).ab,ti,kf.) AND ("Diet, Food, and Nutrition"/ OR exp Food/ OR exp Eating/ OR exp Diet/ OR exp Exercise/ OR exp Body Weight/ OR Dietary Supplements/ OR exp Body Composition/ OR Diet Records/ OR Carbohydrates/ OR Minerals/ OR Vitamins/ OR Nutritional Status/ OR exp Edible Grain/ OR Energy Intake/ OR exp Meat/ OR exp Nuts/ OR exp Vegetables/ OR exp Dairy Products/ OR exp Fruit/ OR "Fruit and Vegetable Juices"/ OR egg/ OR Sugars/ OR Starch/ OR Fatty Acids/ OR Lactation/ OR Breast Feeding/ OR exp Anthropometry/ OR Skinfold Thickness/ OR exp Sports/ OR Sedentary Behavior/ OR Housekeeping/ OR Recreation/ OR Standing Position/ OR Sitting Position/ OR exp Feeding Behavior/ OR Nutrition Assessment/ OR exp Probiotics/ OR exp Prebiotics/ OR Leisure Activities/ OR Physical Functional Performance/ OR exp Body Constitution/ OR Skinfold Thickness/ OR Absorptiometry, Photon/ OR exp Diet Therapy/ OR exp Proteins/ OR Carbonated Beverages/ OR exp Sugar-Sweetened Beverages/ OR Glutens/ OR Caffeine/ OR "Fruit and Vegetable Juices"/ OR Polysaccharides/ OR Sweetening Agents/ OR Vitamin A/ OR Carotenoids/ OR Pyridoxine/ OR Iodine/ OR Phytoestrogens/ OR (nutrition* OR food* OR feeding OR eating OR diet* OR dietary OR supplement OR supplements OR ((physical* OR leisure* OR outdoor*) ADJ3 (activ* OR inactiv*)) OR fitness OR (Physical* ADJ3 (exertion* OR endurance* OR condition* OR Performan* OR education*)) OR Play-time OR chores OR (Planned ADJ3 (exercise)) OR ((skinfold* OR skin-fold*) ADJ3 (thickness OR parameter*)) OR dexa OR bioimpedence OR (Formula ADJ3 (fed OR feading OR baby)) OR soft-drink* OR softdrink* OR ((sweet* OR sugar*) ADJ3 beverage*) OR total-fat OR trans-fat OR gluten OR Fibre* OR Wholegrain* OR wheat* OR Wholewheat* OR soy OR soybean* OR soya OR bread OR milk OR caffein* OR fruit-pulp* OR juice* OR polysaccharid* OR ((fluid* OR water) ADJ3 (intake* OR consum*)) OR drinking OR drink OR drinks OR linol*-acid* OR Folic-acid* OR sweetening OR sweetener* OR hydrogenated-oil* OR retinol* OR carotenoid* OR Niacin OR pyridoxin* OR cobalamin* OR iodine OR phytoestrogen* OR ((hip OR head OR chest) ADJ3 circumfer*) OR bio-impedence OR Adiposity OR (body ADJ3 (weight OR size OR mass OR height OR composition* OR fat OR fatness OR distribut* OR water OR potassium* OR muscle* OR Length)) OR pufa OR mufa OR ((fat OR energy OR iron OR fatty-acid* OR amino-acid* OR protein* OR calcium OR selenium OR magnesium OR potassium OR zinc OR copper OR phosphorus OR salt OR lipid* OR fiber* OR Carbohydrate* OR fish OR mineral* OR vitamin* OR calor* OR meat OR vegetable* OR alcohol OR beverage* OR starch OR grain* OR polyphenol* OR nut OR nuts OR fruit OR dairy OR egg OR eggs OR sugar OR sodium) ADJ3 (consumption OR underconsumption OR overconsumption OR intake OR deficien* OR restrict*)) OR hunger OR sedentary* OR (energ* ADJ3 (expenditure* OR balance* OR density)) OR (muscle ADJ3 (mass* OR weight*)) OR bmi OR (weight ADJ3 (loss OR gain OR reduction*)) OR ((resistance OR strength OR functional) ADJ3 (training)) OR tai-chi OR yoga OR walking OR cycling OR bike-riding OR sports OR sport OR folinic-acid* OR folate OR breastfe* OR breast-fe* OR obes* OR overweight* OR (birth ADJ (size OR weight)) OR (small* ADJ3 (gestation*)) OR birthweight* OR breast-milk OR human-milk OR

phytochemical* OR phyto-chemical* OR lactat* OR anthropomet* OR (waist ADJ3 (hip or circumferen*)) OR ((skinfold OR skin-fold) ADJ3 thickness) OR (weight ADJ3 (loss OR change* OR gain OR reduction*)) OR birth-weight OR birthweight OR exercise OR housekeeping* OR house-keeping* OR recreation* OR video-game* OR videogame* OR (activit* ADJ3 (Household OR Occupation* OR aerobic OR Cardiovascular OR Endurance OR Life-style OR Lifestyle OR Free-living OR Transportation*)) OR (Transportation* ADJ3 (mode)) OR (standing NOT long-standing) OR sitting OR (Television ADJ3 (viewing OR watching OR time)) OR (screen ADJ3 time) OR probiotic* OR prebiotic* OR pro-biotic* OR pre-biotic* OR ascorbic-acid* OR cholecalciferol* OR riboflavin* OR tocopherol* OR thiamin*).ab,ti,kf.) NOT (* Survivors/ OR * Cancer Survivors/ OR (survivor* OR cancer-treat* OR cancer-therap*).ti.) NOT (exp animals/ NOT humans/) NOT (news OR congres* OR abstract* OR book* OR chapter* OR dissertation abstract*).pt.

## Supplementary Text S1C Search terms used for Cochrane CENTRAL register of Trials

Manually deleted abstracts from trial registries

((((cancer* OR neoplas* OR tumor* OR tumour* OR polyp OR polyps OR carcino* OR adenocarcinoma* OR adenoma* OR sarcoma* OR malign*) NEAR/6 (large-intestin* OR colon* OR rectum OR rectal OR colorect* OR desmoid* OR sigmoid* OR gut)) OR ((mass) NEAR/6 (large-intestin* OR colon* OR rectum OR rectal OR colorectal* OR desmoid* OR sygmoid* OR gut) NOT body-mass) OR ((cancer* OR neoplas* OR tumor* OR polyp OR polyps OR mass OR carcino* OR adenocarcinoma* OR adenoma* OR sarcoma* OR malign*) NEAR/3 (bowel*) NOT small-bowel*) OR CRC):Ab,ti) AND ((child* OR infan* OR adolescen* OR preadolescen* OR ((young OR early) NEXT/1 (adult* OR women OR men)) OR prenatal* OR maternal* OR baby OR babies OR newborn* OR (new NEXT/1 born*) OR neonat* OR prematur* OR pre-matur* OR kid OR kids OR toddler* OR teen* OR boy* OR girl* OR minors OR underag* OR (under NEXT/1 (age*)) OR juvenil* OR youth* OR kindergar* OR puber* OR pubescen* OR prepubescen* OR prepubert* OR pediatric* OR paediatric* OR schoolchild* OR highschool* OR primary-school* OR preterm* OR pregnan* OR postnatal* OR post-natal* OR Preschool* OR Pre-school* OR Perinatal* OR Perinatal* OR Sucking OR prenatal* OR antenatal*):ab,ti) AND ((nutrition* OR food* OR feeding OR eating OR diet* OR dietary OR supplement OR supplements OR ((physical* OR leisure* OR outdoor*) NEAR/3 (activ* OR inactiv*)) OR fitness OR (Physical* NEAR/3 (exertion* OR endurance* OR condition* OR Performan* OR education*)) OR Play-time OR chores OR (Planned NEAR/3 (exercise)) OR ((skinfold* OR skin-fold*) NEAR/3 (thickness OR parameter*)) OR dexa OR bioimpedence OR (Formula NEAR/3 (fed OR feading OR baby)) OR soft-drink* OR softdrink* OR ((sweet* OR sugar*) NEAR/3 beverage*) OR total-fat OR trans-fat OR gluten OR Fibre* OR Wholegrain* OR wheat* OR Wholewheat* OR soy OR soybean* OR soya OR bread OR milk OR caffein* OR fruit-pulp* OR juice* OR polysaccharid* OR ((fluid* OR water) NEAR/3 (intake* OR consum*)) OR drinking OR drink OR drinks OR linol* NEXT acid* OR Folic-acid* OR sweetening OR sweetener* OR hydrogenated-oil* OR retinol* OR carotenoid* OR Niacin OR pyridoxin* OR cobalamin* OR iodine OR phytoestrogen* OR ((hip OR head OR chest) NEAR/3 circumfer*) OR bio-impedence OR Adiposity OR (body NEAR/3 (weight OR size OR mass OR height OR composition* OR fat OR fatness OR distribut* OR water OR potassium* OR muscle* OR Length)) OR pufa OR mufa OR ((fat OR energy OR iron OR fatty-acid* OR amino-acid* OR protein* OR calcium OR selenium OR magnesium OR potassium OR zinc OR copper OR phosphorus OR salt OR lipid* OR fiber* OR Carbohydrate* OR fish OR mineral* OR

vitamin* OR calor* OR meat OR vegetable* OR alcohol OR beverage* OR starch OR grain* OR polyphenol* OR nut OR nuts OR fruit OR dairy OR egg OR eggs OR sugar OR sodium) NEAR/3 (consumption OR underconsumption OR overconsumption OR intake OR deficien* OR restrict*)) OR hunger OR sedentary* OR (energ* NEAR/3 (expenditure* OR balance* OR density)) OR (muscle NEAR/3 (mass* OR weight*)) OR bmi OR (weight NEAR/3 (loss OR gain OR reduction*)) OR ((resistance OR strength OR functional) NEAR/3 (training)) OR tai-chi OR yoga OR walking OR cycling OR bike-riding OR sports OR sport OR folinic-acid* OR folate OR breastfe* OR breast-fe* OR obes* OR overweight* OR (birth NEXT/1 (size OR weight)) OR (small* NEAR/3 (gestation*)) OR birthweight* OR breast-milk OR human-milk OR phytochemical* OR phyto-chemical* OR lactat* OR anthropomet* OR (waist NEAR/3 (hip or circumferen*)) OR ((skinfold OR skin-fold) NEAR/3 thickness) OR (weight NEAR/3 (loss OR change* OR gain OR reduction*)) OR birth-weight OR birthweight OR exercise OR housekeeping* OR house-keeping* OR recreation* OR video-game* OR videogame* OR (activit* NEAR/3 (Household OR Occupation* OR aerobic OR Cardiovascular OR Endurance OR Life-style OR Lifestyle OR Free-living OR Transportation*)) OR (Transportation* NEAR/3 (mode)) OR (standing NOT long-standing) OR sitting OR (Television NEAR/3 (viewing OR watching OR time)) OR (screen NEAR/3 time) OR probiotic* OR prebiotic* OR pro-biotic* OR pre-biotic* OR ascorbic-acid* OR cholecalciferol* OR riboflavin* OR tocopherol* OR thiamin*):ab,ti) NOT ((survivor* OR cancer-treat* OR cancer-therap*):ti) NOT "conference abstract":pt

## Supplementary Text S1D Search terms used for Web of Science Core Collection

Science Citation Index Expanded (1975-present) ; Social Sciences Citation Index (1975-present) ; Arts & Humanities Citation Index (1975-present) ; Conference Proceedings Citation Index- Science (1990-present) ; Conference Proceedings Citation Index- Social Science & Humanities (1990-present) ; Emerging Sources Citation Index (2005-present)

TS=(((((cancer* OR neoplas* OR tumor* OR tumour* OR polyp OR polyps OR carcino* OR adenocarcinoma* OR adenoma* OR sarcoma* OR malign*) NEAR/5 (large-intestin* OR colon* OR rectum OR rectal OR colorect* OR desmoid* OR sigmoid* OR gut)) OR ((mass) NEAR/5 (large-intestin* OR colon* OR rectum OR rectal OR colorectal* OR desmoid* OR sygmoid* OR gut) NOT body-mass) OR ((cancer* OR neoplas* OR tumor* OR polyp OR polyps OR mass OR carcino* OR adenocarcinoma* OR adenoma* OR sarcoma* OR malign*) NEAR/2 (bowel*) NOT small-bowel*) OR CRC)) AND ((child* OR infan* OR adolescen* OR preadolescen* OR ((young OR early) NEAR/1 (adult* OR women OR men)) OR prenatal* OR maternal* OR baby OR babies OR newborn* OR (new NEAR/1 born*) OR neonat* OR prematur* OR pre-matur* OR kid OR kids OR toddler* OR teen* OR boy* OR girl* OR minors OR underag* OR (under NEAR/1 (age*)) OR juvenil* OR youth* OR kindergar* OR puber* OR pubescen* OR prepubescen* OR prepubert* OR pediatric* OR paediatric* OR schoolchild* OR highschool* OR primary-school* OR preterm* OR pregnan* OR postnatal* OR post-natal* OR Preschool* OR Pre-school* OR Perinatal* OR Perinatal* OR Sucking OR prenatal* OR antenatal*)) AND ((nutrition* OR food* OR feeding OR eating OR diet* OR dietary OR supplement OR supplements OR ((physical* OR leisure* OR outdoor*) NEAR/2 (activ* OR inactiv*)) OR fitness OR (Physical* NEAR/2 (exertion* OR endurance* OR condition* OR Performan* OR education*)) OR Play-time OR chores OR (Planned NEAR/2 (exercise)) OR ((skinfold* OR skin-fold*) NEAR/2 (thickness OR parameter*)) OR dexa OR bioimpedence OR (Formula NEAR/2 (fed OR feading OR baby)) OR soft-drink* OR softdrink* OR ((sweet* OR sugar*) NEAR/2 beverage*) OR total-fat OR trans-fat OR gluten OR Fibre* OR Wholegrain* OR wheat* OR Wholewheat* OR soy OR soybean* OR soya OR bread OR milk OR caffein*

OR fruit-pulp* OR juice* OR polysaccharid* OR ((fluid* OR water) NEAR/2 (intake* OR consum*)) OR drinking OR drink OR drinks OR linol*-acid* OR Folic-acid* OR sweetening OR sweetener* OR hydrogenated-oil* OR retinol* OR carotenoid* OR Niacin OR pyridoxin* OR cobalamin* OR iodine OR phytoestrogen* OR ((hip OR head OR chest) NEAR/2 circumfer*) OR bio-impedence OR Adiposity OR (body NEAR/2 (weight OR size OR mass OR height OR composition* OR fat OR fatness OR distribut* OR water OR potassium* OR muscle* OR Length)) OR pufa OR mufa OR ((fat OR energy OR iron OR fatty-acid* OR amino-acid* OR protein* OR calcium OR selenium OR magnesium OR potassium OR zinc OR copper OR phosphorus OR salt OR lipid* OR fiber* OR Carbohydrate* OR fish OR mineral* OR vitamin* OR calor* OR meat OR vegetable* OR alcohol OR beverage* OR starch OR grain* OR polyphenol* OR nut OR nuts OR fruit OR dairy OR egg OR eggs OR sugar OR sodium) NEAR/2 (consumption OR underconsumption OR overconsumption OR intake OR deficien* OR restrict*)) OR hunger OR sedentary* OR (energ* NEAR/2 (expenditure* OR balance* OR density)) OR (muscle NEAR/2 (mass* OR weight*)) OR bmi OR (weight NEAR/2 (loss OR gain OR reduction*)) OR ((resistance OR strength OR functional) NEAR/2 (training)) OR tai-chi OR yoga OR walking OR cycling OR bike-riding OR sports OR sport OR folinic-acid* OR folate OR breastfe* OR breast-fe* OR obes* OR overweight* OR (birth NEAR/1 (size OR weight)) OR (small* NEAR/2 (gestation*)) OR birthweight* OR breast-milk OR human-milk OR phytochemical* OR phyto-chemical* OR lactat* OR anthropomet* OR (waist NEAR/2 (hip or circumferen*)) OR ((skinfold OR skin-fold) NEAR/2 thickness) OR (weight NEAR/2 (loss OR change* OR gain OR reduction*)) OR birth-weight OR birthweight OR exercise OR housekeeping* OR house-keeping* OR recreation* OR video-game* OR videogame* OR (activit* NEAR/2 (Household OR Occupation* OR aerobic OR Cardiovascular OR Endurance OR Life-style OR Lifestyle OR Free-living OR Transportation*)) OR (Transportation* NEAR/2 (mode)) OR (standing NOT long-standing) OR sitting OR (Television NEAR/2 (viewing OR watching OR time)) OR (screen NEAR/2 time) OR probiotic* OR prebiotic* OR pro-biotic* OR pre-biotic* OR ascorbic-acid* OR cholecalciferol* OR riboflavin* OR tocopherol* OR thiamin*))) NOT TI=((survivor* OR cancer-treat* OR cancer-therap*)) NOT DT=(Meeting Abstract OR Meeting Summary)

## Supplementary Table S1 PRISMA 2020 Checklist

| Section and Topic | Item # | Checklist item | Location where item is reported |
|---|---|---|---|
| **TITLE** | | | |
| Title | 1 | Identify the report as a systematic review. | Title |
| **ABSTRACT** | | | |
| Abstract | 2 | See the PRISMA 2020 for Abstracts checklist. | |
| **INTRODUCTION** | | | |
| Rationale | 3 | Describe the rationale for the review in the context of existing knowledge. | Introduction |
| Objectives | 4 | Provide an explicit statement of the objective(s) or question(s) the review addresses. | Introduction, last paragraph |
| **METHODS** | | | |
| Eligibility criteria | 5 | Specify the inclusion and exclusion criteria for the review and how studies were grouped for the syntheses. | Study selection |
| Information sources | 6 | Specify all databases, registers, websites, organisations, reference lists and other sources searched or consulted to identify studies. Specify the date when each source was last searched or consulted. | Search strategy |
| Search strategy | 7 | Present the full search strategies for all databases, registers and websites, including any filters and limits used. | Supplementary text S1 |
| Selection process | 8 | Specify the methods used to decide whether a study met the inclusion criteria of the review, including how many reviewers screened each record and each report retrieved, whether they worked independently, and if applicable, details of automation tools used in the process. | Study selection |
| Data collection process | 9 | Specify the methods used to collect data from reports, including how many reviewers collected data from each report, whether they worked independently, any processes for obtaining or confirming data from study investigators, and if applicable, details of automation tools used in the process. | Data extraction |
| Data items | 10a | List and define all outcomes for which data were sought. Specify whether all results that were compatible with each outcome domain in each study were sought (e.g. for all measures, time points, analyses), and if not, the methods used to decide which results to collect. | Study selection & Data extraction |
| | 10b | List and define all other variables for which data were sought (e.g. participant and intervention characteristics, funding sources). Describe any assumptions made about any missing or unclear information. | Data extraction & Supplementary Tables S2-6 |
| Study risk of bias assessment | 11 | Specify the methods used to assess risk of bias in the included studies, including details of the tool(s) used, how many reviewers assessed each study and whether they worked independently, and if applicable, details of automation tools used in the process. | Risk of bias assessment & Supplementary Tables S2-6 |

| Section and Topic | Item # | Checklist item | Location where item is reported |
|---|---|---|---|
| Effect measures | 12 | Specify for each outcome the effect measure(s) (e.g. risk ratio, mean difference) used in the synthesis or presentation of results. | Statistical analyses paragraph 1 |
| Synthesis methods | 13a | Describe the processes used to decide which studies were eligible for each synthesis (e.g. tabulating the study intervention characteristics and comparing against the planned groups for each synthesis (item #5)). | Study selection & Statistical analyses |
| | 13b | Describe any methods required to prepare the data for presentation or synthesis, such as handling of missing summary statistics, or data conversions. | Study selection paragraph 2 & Statistical analyses paragraph 2 |
| | 13c | Describe any methods used to tabulate or visually display results of individual studies and syntheses. | Statistical analyses |
| | 13d | Describe any methods used to synthesize results and provide a rationale for the choice(s). If meta-analysis was performed, describe the model(s), method(s) to identify the presence and extent of statistical heterogeneity, and software package(s) used. | Statistical analyses |
| | 13e | Describe any methods used to explore possible causes of heterogeneity among study results (e.g. subgroup analysis, meta-regression). | Statistical analyses, last paragraph |
| | 13f | Describe any sensitivity analyses conducted to assess robustness of the synthesized results. | Statistical analyses, last paragraph |
| Reporting bias assessment | 14 | Describe any methods used to assess risk of bias due to missing results in a synthesis (arising from reporting biases). | Statistical analyses, second paragraph |
| Certainty assessment | 15 | Describe any methods used to assess certainty (or confidence) in the body of evidence for an outcome. | Evidence grading criteria & Supplementary Table S7 |
| **RESULTS** | | | |
| Study selection | 16a | Describe the results of the search and selection process, from the number of records identified in the search to the number of studies included in the review, ideally using a flow diagram. | Supplementary Figure S1 & |

| Section and Topic | Item # | Checklist item | Location where item is reported |
|---|---|---|---|
| | | | Results, first paragraph |
| | 16b | Cite studies that might appear to meet the inclusion criteria, but which were excluded, and explain why they were excluded. | Results, first paragraph & Supplementary Table S8 |
| Study characteristics | 17 | Cite each included study and present its characteristics. | Supplementary Table S2-6, S9-10 |
| Risk of bias in studies | 18 | Present assessments of risk of bias for each included study. | Supplementary Table S2-6, S9 & Results, second paragraph |
| Results of individual studies | 19 | For all outcomes, present, for each study: (a) summary statistics for each group (where appropriate) and (b) an effect estimate and its precision (e.g. confidence/credible interval), ideally using structured tables or plots. | Supplementary Table S2-6, S9-10 |
| Results of syntheses | 20a | For each synthesis, briefly summarise the characteristics and risk of bias among contributing studies. | Results, first and second paragraph |
| | 20b | Present results of all statistical syntheses conducted. If meta-analysis was done, present for each the summary estimate and its precision (e.g. confidence/credible interval) and measures of statistical heterogeneity. If comparing groups, describe the direction of the effect. | Results & Figure 1-4 & Table 2 |
| | 20c | Present results of all investigations of possible causes of heterogeneity among study results. | Consistency of results & Table 1 |
| | 20d | Present results of all sensitivity analyses conducted to assess the robustness of the synthesized results. | Consistency of results |
| Reporting biases | 21 | Present assessments of risk of bias due to missing results (arising from reporting biases) for each synthesis assessed. | Young adult adiposity |
| Certainty of evidence | 22 | Present assessments of certainty (or confidence) in the body of evidence for each outcome assessed. | Evidence grading & Table 2 |
| **DISCUSSION** | | | |

| Section and Topic | Item # | Checklist item | Location where item is reported |
|---|---|---|---|
| Discussion | 23a | Provide a general interpretation of the results in the context of other evidence. | Discussion, first three paragraphs |
| | 23b | Discuss any limitations of the evidence included in the review. | Discussion, fourth paragraph |
| | 23c | Discuss any limitations of the review processes used. | Discussion, fourth paragraph |
| | 23d | Discuss implications of the results for practice, policy, and future research. | Conclusion, last paragraph |
| **OTHER INFORMATION** | | | |
| Registration and protocol | 24a | Provide registration information for the review, including register name and registration number, or state that the review was not registered. | Methods, first paragraph |
| | 24b | Indicate where the review protocol can be accessed, or state that a protocol was not prepared. | Methods, first paragraph |
| | 24c | Describe and explain any amendments to information provided at registration or in the protocol. | Methods, first paragraph |
| Support | 25 | Describe sources of financial or non-financial support for the review, and the role of the funders or sponsors in the review. | Funding information |
| Competing interests | 26 | Declare any competing interests of review authors. | Conflict of interest statement |
| Availability of data, code and other materials | 27 | Report which of the following are publicly available and where they can be found: template data collection forms; data extracted from included studies; data used for all analyses; analytic code; any other materials used in the review. | Data availability statement |

*From:* Page MJ, McKenzie JE, Bossuyt PM, Boutron I, Hoffmann TC, Mulrow CD, et al. The PRISMA 2020 statement: an updated guideline for reporting systematic reviews. BMJ 2021;372:n71. doi: 10.1136/bmj.n71

For more information, visit: http://www.prisma-statement.org/

## Supplementary Table S2 Results from studies examining the association between birth size and risk of colorectal cancer in adulthood

| | | | | | | | | | NOS QS | | | |
|---|---|---|---|---|---|---|---|---|---|---|---|---|
| Author, Year, Country | Study name (study design), Characteristics | Cases / Study size | Length of follow-up, age at end follow-up or CRC diagnosis (range), case ascertainment | Exposure assessment / age at exposure assessment | Subsite (sex) | Comparison | RR (95% CI) P-trend | Adjustment factors | Selection | Comparability | Outcome | Total |
| *Intra-uterine life (from conception until birth, including measurements at birth) - Birthweight* | | | | | | | | | | | | |
| Murphy 2022 (1), USA | Child Health and Development Studies (HCDS) (cohort), men and women born in 1959-1967. | 68 / 18,751 | Followed from birth to 2019 (max 58.5 y). Age range at diagnosis 18-56 y, 49% diagnosed <50 y. Cancer registry | Birth weight measured by research staff | CRC | <2500 g vs 2500-3999 g | 0.44 (0.06-3.15) | Race/ethnicity, maternal BMI, maternal rate of early weight gain during pregnancy, maternal total weight gain during pregnancy. | * * * * | * | * * * | 8 |
| | | | | | | ≥4000 g vs 2500-3999 g | 1.95 (0.86-4.38) | | | | | |
| Smith 2016 (2), Denmark | Copenhagen School Health Record Register (CSHRR) (cohort), men and women born in 1936-1972. | 2571 / 193,306 | Followed from 1978 or 40th birthday to 2012 (max 34 y). Cancer registry | Birth weight reported by parents during health examination at school entry at age 5-7 y | CRC | Per kg | 1.05 (0.98-1.13) | Age (as time axis). Stratification factors: birth cohort and sex. | * * * | * | * * * | 7 |
| | | 1465 / 193,306 | | | CC | Per kg | 1.14 (1.04-1.26) | | | | | |
| | | 961 / 193,306 | | | RC | 2,500 g vs 3,500 g | 0.92 (0.76-1.12) | | | | | |
| | | | | | | 2,500 g vs 3,500 g | 0.77 (0.61-0.96) | | | | | |
| Spracklen 2014 (3), USA | Women's Health Initiative Observational Study (WHI-OS) (cohort), post-menopausal women cancer-free at age 50-79 y (mean 63 y) in 1993-1998. Women were excluded when born premature. | 625 / 60,848 | Followed from age 50-79 y for 11.3 y. Cancer: self-reported verified by medical records and pathology reports. The National Death Index was periodically searched to identify deaths of participants lost to follow-up. | Birth weight self-reported at age 50-79 y | CC (F) | <6 lbs vs 6-7.9 lbs | 0.75 (0.53-1.05) | Age, race/ethnicity, education, and socioeconomic status. | * * | * * | * * * | 7 |
| | | | | | | 8-9.9 lbs vs 6-7.9 lbs | 1.28 (1.06-1.54) | | | | | |
| | | | | | | ≥10 lbs vs 6-7.9 lbs | 0.83 (0.53-1.32) | | | | | |
| | | | | | | | P for trend 0.03 | | | | | |
| Yang 2014 (4), England & Scotland | Million Women Study (MWS)(cohort), women born in the 1930s and 1940s and cancer-free at age 50-64 y (mean 59 y) in 1996-2001. | 4414 / 453,023 | Followed from age 53-67 y until 2011 (mean 9.2 y). Cancer registry | Birth weight self-reported at age 53-67 y | CRC (F) | Per kg | 1.09 (0.99-1.20) | Age (as time axis). Stratification factors: year of birth, region of residence. Additional adjustment for maternal smoking during pregnancy, | * * | * | * * | 5 |

| | | | | | | | | | | | | |
|---|---|---|---|---|---|---|---|---|---|---|---|---|
| | | | | | | | | having been breast fed as an infant, adult smoking, adult BMI, adult strenuous exercise or adult alcohol consumption did not change the RR and 95% CI. | | | | |
| Barker 2013 (5), Finland | Helsinki Birth Cohort Study (HBCS) (cohort), men and women born in 1924-1944. | 275 / 20,431 | Followed from birth until 2006. Mean age at diagnosis 63 y (28-79 y). Cancer registry | Birth weight from birth record. | CRC | Per kg | 1.1 (0.8 - 1.3) | Stratification factors: Sex, year of birth. | * * * * | * | * * * | 8 |
| Cnattingius 2009 (6), Sweden | Swedish Twin Registry (STR)(cohort), twin men and women born in 1926-1958. | 248 / 23,337 | Followed in 1973-2006 (max 34 y), age range at end of follow-up 48-80 y. Cancer registry | Birth weight from birth record | CRC | <2500 g vs 2500-2999 g | 1.07 (0.69-1.64) | Age (as time axis), zygosity, sex, gestational age, maternal age, parity, and maternal socioeconomic status at birth. Stratification factor: birth year. | * * * | * * | * * * | 8 |
| | | | | | | ≥3000 g vs 2500-2999 g | 1.04 (0.66-1.64) | | | | | |
| McCormak 2005 (7), Sweden | Uppsala Birth Cohort Study (UBoS) (cohort), men and women born in 1915-1929. | 259 / 11,166 | Followed from 1960 (median age 37 y) to 2001 (max 41 y). Cancer registry | Birth weight from detailed obstetric notes | CRC | Per 1 SD (450 g) | 1.16 (1.02-1.33) | Age (as time axis), sex, birth order, maternal marital status, maternal social class at birth, and socioeconomic factors during adulthood. Stratification factor: birth year. | * * * * | * | * * * | 8 |
| Nilsen 2005 (8), Norway | St Olav's University hospital (cohort), men and women born in 1920-1958. | 247 / 35,697 | Followed from 1961 or 20th birthday to 2001, median 31 y (max 41 y). Median age at diagnosis was 59 y (range 26-79 y). Cancer registry | Birth weight from birth record | CRC (M) | <3270 g vs ≥3925 g | 1.7 (1.0-2.8) | Birth year, maternal age at childbearing, length of gestation, gestational hypertension and/or pre-eclampsia, birth order, maternal height, maternal marital status, and maternal socioeconomic status. | * * * * | * * | * * * | 9 |
| | | | | | | 3270-3590 g vs ≥3925 g | 1.3 (0.7-2.2) | | | | | |
| | | | | | | 3595-3920 g vs ≥3925 g | 1.8 (1.1-2.9) | | | | | |
| | | | | | | | P-trend 0.20 | | | | | |
| | | | | | CRC (F) | <3150 g vs ≥3765 g | 1.3 (0.7-2.4) | | | | | |
| | | | | | | 3150-3440 g vs ≥3765 g | 0.7 (0.4-1.4) | | | | | |

| | | | | | | | | | | | | |
|---|---|---|---|---|---|---|---|---|---|---|---|---|
| | | | | | | 3445-3760 g vs ≥3765 g | 1.2 (0.7-2.2) | | | | | |
| | | | | | | | P-trend 0.65 | | | | | |
| Sandhu 2002 (9), UK | EPIC Norfolk (cohort), cancer-free men and women (62%) aged 45-79 in 1993-1997. | 52 / 11,805 | Followed from age 45-79 y for mean >4 y. Cancer registry | Birth weight self-reported at age 45-79 y | CRC | <2500 g vs 2500-3249 g | 2.16 (0.89-5.22) | Age (as time axis) and sex. | * * | | * * | 4 |
| | | | | | | 3250-4000 g vs 2500-3249 g | 1.83 (0.87-3.86) | | | | | |
| | | | | | | >4000 g vs 2500-3249 g | 2.57 (1.15-5.74) | | | | | |
| *Intra-uterine life (from conception until birth, including measurements at birth) – Birth length* | | | | | | | | | | | | |
| Cnattingius 2009 (6), Sweden | Swedish Twin Registry (STR)(cohort), twin men and women (51%) born in 1926-1958. | 248 / 23,337 | Followed in 1973-2006 (max 34 y), age range at end of follow-up 48-80 y. Cancer registry | Birth length from birth record | CRC | <47 cm vs 47-49 cm | 0.84 (0.59-1.20) | Age (as time axis), zygosity, sex, gestational age, maternal age, parity, and maternal socioeconomic status at birth. Stratification factor: birth year. | * * * | * * | * * * | 8 |
| | | | | | | ≥ 50 cm vs 47-49 cm | 1.21 (0.89-1.65) | | | | | |
| McCormak 2005 (7), Sweden | Uppsala Birth Cohort Study (UBoS) (cohort), men and women (48%) born in 1915-1929. | 259 / 11,166 | Followed from 1960 (median age 37 y) to 2001 (max 41 y). Cancer registry | Birth length from detailed obstetric notes | CRC | Per 1 SD (2.0 cm) | 1.04 (0.91-1.19) | Age (as time axis), sex, birth order, maternal marital status, maternal social class at birth, and socioeconomic factors during adulthood. Stratification factor: birth year. | * * * * | * | * * * | 8 |
| Nilsen 2005 (8), Norway | St Olav's University hospital (cohort), men and women (45%) born in 1920-1958. | 247 / 35,697 | Followed from 1961 or 20th birthday to 2001, median 31 y (max 41 y). Median age at diagnosis was 59 y (range 26-79 y). Cancer registry | Birth length from birth record | CRC (M) | <51 cm vs ≥ 53 cm | 1.9 (1.0-3.7) | Birth year, maternal age at childbearing, length of gestation, gestational hypertension and/or pre-eclampsia, birth order, maternal height, maternal marital status, and maternal socioeconomic status. | * * * * | * * | * * * | 9 |
| | | | | | | 51 cm vs ≥ 53 cm | 2.1 (1.1-4.1) | | | | | |
| | | | | | | 52 cm vs ≥ 53 cm | 1.3 (0.6-2.7) | | | | | |
| | | | | | | | P-trend 0.03 | | | | | |
| | | | | | CRC (F) | <50 cm vs ≥ 52 cm | 0.7 (0.4-1.3) | | | | | |
| | | | | | | 50 cm vs ≥ 52 cm | 0.7 (0.4-1.2) | | | | | |

| | | | | | | | | | | | | |
|---|---|---|---|---|---|---|---|---|---|---|---|---|
| | | | | | | 51 cm vs ≥ 52 cm | 0.7 (0.3-1.4) | | | | | |
| | | | | | | | P-trend 0.33 | | | | | |

Abbreviations: CC, colon cancer; CRC, colorectal cancer; F, female; M, male; RC, rectal cancer. NOS QS (NOS quality score): For selection, four stars could be awarded based on being: (1) representativeness of the exposed cohort; (2) selection of the non-exposed cohort; (3) ascertainment of exposure from a secure record, measured objectively, or a validated self-report method; and (4) demonstration that CRC was not present at the start of the study. For comparability, studies could score one star when they controlled for sex (if applicable) and gestational age. Another star was awarded when the study controlled for age/birth year, socio-economic status, race/ethnicity, maternal smoking (status), or maternal alcohol intake. For outcome, 3 stars could be awarded: (1) outcome assessment through linkage with a cancer registry or verified self-report; (2) when the follow-up period was 10 years or more and the mean age of the population at diagnosis was at least 50 years (to assure sufficient cancer cases when follow-up started during early life); (3) follow-up rate ≥ 80% or outcome assessment through linkage with a cancer registry.

## Supplementary Table S3 Results from studies examining the association between childhood body fatness and height and risk of colorectal cancer in adulthood

| | | | | | | | | | NOS QS | | | |
|---|---|---|---|---|---|---|---|---|---|---|---|---|
| Author, Year, Country | Study name (study design), Characteristics | Cases / Study size | Length of follow-up, age at end follow-up or CRC diagnosis (range), case ascertainment | Exposure assessment / age at exposure assessment | Subsite (sex) | Comparison | RR (95% CI) P-trend | Adjustment factors | Selection | Comparabilit | Outcome | |
| *Childhood BMI (from 2 until <10 years)* | | | | | | | | | | | | |
| Celind 2019 (10), Sweden | Gothenburg (cohort), men born in 1946 to 1961. | 257 / 37,663 | Followed from age 20 y to 2013 (mean 37.7 y). Cancer registry. | Measured BMI between 6.5-9.5 y (age-adjusted to 8 y). Overweight was defined according to the CDC cut-off (BMI ≥ 17.9 kg/m²). | CC (M) | Per SD (1.4 kg/m²) | 1.19 (1.06-1.33) | Birth year and country of birth. | **** | ** | *** | 9 |
| | | | | | | Overweight vs non-overweight | 1.78 (1.19-2.67) | | | | | |
| | | 159 / 37,663 | | | RC (M) | Per SD (1.4 kg/m²) | 0.99 (0.85-1.16) | | | | | |
| | | | | | | Overweight vs non-overweight | 1.17 (0.63-2.16) | | | | | |
| Jensen 2017 (11), Denmark | Copenhagen School Health Records Register (CSHRR) (cohort), men and women born in 1930 to 1972. Cancer-free in 1978. | 2,487 / 242,939 | Followed from 1978 or 40 y to 2012 (mean 21 y). Age range at diagnosis 40-83 y. Cancer registry. | Measured BMI at 7 y | CC | Per z-score | 1.07 (1.02-1.12) | Age (as time axis), birth cohort, and sex. | **** | ** | *** | 9 |
| | | 1,553 / 242,939 | | | RC | Per z-score | 0.96 (0.91-1.02) | | | | | |
| Jeffreys 2004 (12), England and Scotland | Boyd Orr (cohort), men and women born in the 1920s-1930s. | 38 / 2,347 | Followed from 1937/39 (mean age 8 y) to 2001/03 (max 60 y). Cancer registry and death certificates. | Measured BMI between 2-14.8 y (mean 8 y). Very lean cohort; only 3.8% were over the 90th percentile of contemporary BMI standards. | CRC | Per z-score | 0.96 (0.70-1.32) | Age and sex. | *** | ** | *** | 8 |
| *Childhood BMI pictograms (from 2 until <10 years)* | | | | | | | | | | | | |
| Li 2017 (13), Sweden | Karolinska Mammography (KARMA) (cohort), women with a mean age of 55 y at study entry in 2011-2013. | 235 / 65,057 | Followed from age 7 to 2013. Average age at diagnosis 55 (SD 14) y. Cancer registry. | Self-reported body fatness at 7 y using the Stunkard Figure Rating Scale / Mean age 55 y | CC (F) | Per pictogram | 1.05 (0.96-1.15) | Age (as time axis) and birth cohort. | *** | ** | *** | 8 |
| Zhang 2015 (14), USA | Nurses' Health Study (NHS) (cohort), women aged 30-55 y in 1976. Cancer-free in 1988 (mean age 55 y). Women were excluded when they had ulcerative colitis. | 1,292 / 75,238 | Followed from 1988 (mean age 55/56 y) to 2010 (22 y). Self-reported cancer verified by medical records. | Self-reported average body fatness at age 5 and 10 using the Stunkard Figure Rating Scale / Mean age 55 (F) or 56 (M) y. | CRC | Overweight (pictogram 5+) vs most lean (pictogram 1) | 1.32 (1.08-1.31) | Age, height, smoking before age 30, family history of CRC, history of sigmoidoscopy/endoscopy, current | *** | ** | *** | 8 |

| | | | | | | | | | | | | |
|---|---|---|---|---|---|---|---|---|---|---|---|---|
| | Health Professionals Follow-up Study (HPFS) (cohort), men aged 40-75 y in 1986. Cancer-free in 1988 (mean age 56 y). Men were excluded when they had ulcerative colitis. | 808 / 34,533 | | | | | 1.12 (0.90-1.59) | physical activity, aspirin use, processed meat, and postmenopausal hormone use (women only). | * * * | * * | * * * | 8 |
| *Childhood height (from 2 until <10 years)* | | | | | | | | | | | | |
| Celind 2019 (10), Sweden | Gothenburg (cohort), men born in 1946 to 1961. | 257 / 37,663 | Followed from age 20 y to 2013 (mean 37.7 y). Cancer registry. | Measured height between 6.5-9.5 y (age-adjusted to 8 y). | CC (M) | Per SD | 1.05 (0.93-1.18) | Birth year and country of birth. | * * * * | * * | * * * | 9 |
| | | 159 / 37,663 | | | RC (M) | Per SD | 1.02 (0.87-1.19) | | | | | |
| Jensen 2017 (11), Denmark | Copenhagen School Health Records Register (CSHRR) (cohort), men and women born in 1930 to 1972. Cancer-free in 1978. | 2,487 / 242,939 | Followed from 1978 or 40 y to 2012 (mean 21 y). Age range at diagnosis 40-83 y. Cancer registry. | Measured height at 7 y | CC | Per z-score | 1.13 (1.09-1.18) | Age (as time axis), birth cohort, and sex. | * * * * | * * | * * * | 9 |
| | | 1,553 / 242,939 | | | RC | Per z-score | 1.02 (0.97-1.08) | | | | | |
| Whitley 2009 (15), England & Scotland | Boyd Orr (cohort), men and women born in the 1920s-1930s. | 59 / 2,969 | Followed from 1937/39 (mean age 8 y) to 2001/03 (max 60 y). Cancer registry and death certificates. | Measured height between 2-14.8 y (mean 8 y). | CRC | Per SD | 1.06 (0.84-1.33) | Age and sex. | * * * | * * | * * * | 8 |

Abbreviations: CC, colon cancer; CRC, colorectal cancer; F, female; M, male; RC, rectal cancer. NOS QS (NOS quality score): For selection, four stars could be awarded based on being: (1) representativeness of the exposed cohort; (2) selection of the non-exposed cohort; (3) ascertainment of exposure from a secure record, measured objectively, or a validated self-report method; and (4) demonstration that CRC was not present at the start of the study. For comparability, studies could score one star when they controlled for sex (if applicable). Another star was awarded when the study controlled for age/birth year, socio-economic status, race/ethnicity, parental smoking (status), or childhood physical activity. For outcome, 3 stars could be awarded: (1) outcome assessment through linkage with a cancer registry or verified self-report; (2) when the follow-up period was 10 years or more and the mean age of the population at diagnosis was at least 50 years (to assure sufficient cancer cases when follow-up started during early life); (3) follow-up rate ≥ 80% or outcome assessment through linkage with a cancer registry.

## Supplementary Table S4 Results from studies examining the association between adolescent body fatness and height and risk of colorectal cancer in adulthood

| | | | | | | | | | NOS QS | | | |
|---|---|---|---|---|---|---|---|---|---|---|---|---|
| Author, Year, Country | Study name (study design), Characteristics | Cases / Study size | Length of follow-up, age at start and/or end follow-up or CRC diagnosis (range), case ascertainment | Exposure assessment, / age at exposure assessment | Subsite | Comparison | RR (95% CI) P-trend | Adjustment factors | Selection | Comparabil | Outcome | |
| *Adolescent BMI (from 10 until 19 years)* | | | | | | | | | | | | |
| Furer 2020 (16), Israel | Israel (cohort), cancer-free men and women at military recruitment assessment (age ~17 y) in 1967-1996. | 1,231 / 1,370,020 | Followed from age 17 y until 2012 (mean ~20 y). Mean age at diagnosis was ~48 y for men and ~45 y for women. Cancer registry. | Measured BMI at age 16-19 y (mean 17.4 y, SD 0.4). Overweight (≥ 85th percentile) and obesity ((≥ 95th percentile) were defined according to the CDC cut-offs. | CC (M) | Per 5 $kg/m^2$ | 1.29 (1.17-1.42) | Age, birth year, sex, socioeconomic status, education, and region of origin. | * * * * | * * | * * | 8 |
| | | 660 / 928,110 | | | CC (F) | Per 5 $kg/m^2$ | 1.17 (1.03-1.33) | | | | | |
| | | 783 / 1,370,020 | | | RC (M) | Per 5 $kg/m^2$ | 1.04 (0.92-1.17) | | | | | |
| | | 350 / 928,110 | | | RC (F) | Per 5 $kg/m^2$ | 1.16 (0.98-1.38) | | | | | |
| Jensen 2017 (11), Denmark | Copenhagen School Health Records Register (CSHRR) (cohort), men and women born in 1930 to 1972. Cancer-free in 1978. | 2,487 / 242,939 | Followed from 1978 or age 40 y to 2012 (mean 21.1 y). Age range at diagnosis 40-83 y. Cancer registry. | Measured BMI at age 13 y. | CC | Per z-score | 1.09 (1.04-1.14) | Age (as time axis), birth cohort, and sex. | * * * * | * * | * * * | 9 |
| | | 1,553 / 242,939 | | | RC | Per z-score | 0.97 (0.92-1.03) | | | | | |
| Batty 2015 (17), Scotland | Scottish Mental Survey 1947 (SMS1947) (cohort), men and women born in 1936 attending school at study entry (age 11 y). | 127 / 4,620 | Followed from 1947 (age 11 y) until 2014 (max 67 y). Cancer registry. | Measured BMI at age 11 y. Very lean cohort by contemporary standards. | CRC | Per 1 SD | 1.01 (0.85-1.20) | Age (as time axis), sex, fathers' occupation, occupancy rate, height, and physical disability. | * * * | * * | * * * | 8 |
| Must 1992 (18), USA | Third Harvard Growth Study (3rd HGS) (cohort), men attending first- or second-grade public-school at study entry in 1922-1923. | 5 / 256 | Followed until 1988 (max 55 y). Mean age of surviving men at end of follow-up 73 y. Self-reported cancer. | Annually measured BMI age 13-18 y. Overweight was defined as a BMI >75th percentile for any two years; the lean group had a BMI that remained between 25th-50th percentile. | CRC (M) | >75th percentile vs 25th-50th percentile | 6.5 (0.7-57.9) | NA | * * * | * | * | 5 |
| *Adolescent BMI pictograms (from 10 until ~19 years)* | | | | | | | | | | | | |
| Zhang 2015 (14), USA | Nurses' Health Study (NHS) (cohort), women aged 30-55 y in 1976. Cancer-free in 1988 (mean | 1,292 / 75,238 | Followed from 1988 (mean age 55/56 y) to 2010 (22 y). | Self-reported average body fatness at age 10 and 20 using the | CRC | Overweight (pictogram 5+) | 1.33 (1.06-1.67) | Age, height, smoking before age 30, family history of CRC, history of sigmoidoscopy/endoscopy, | * * * | * * | * * * | 8 |

| | | | | | | | | | | | | |
|---|---|---|---|---|---|---|---|---|---|---|---|---|
| | age 55 y). Women were excluded when they had ulcerative colitis. | | Self-reported cancer verified by medical records. | Stunkard Figure Rating Scale / Mean age 55 (F) or 56 (M) y. | | vs most lean (pictogram 1) | | current physical activity, aspirin use, processed meat, and postmenopausal hormone use (women only). | | | | |
| | Health Professionals Follow-Up Study (HPFS) (cohort), man aged 40-75 y in 1986. Cancer-free in 1988 (mean age 56 y). Men were excluded when they had ulcerative colitis. | 808 / 34,533 | | | | | 1.11 (0.86-1.44) | | * * * | * * | * * * | 8 |
| *Adolescent height (from 10 until 19 years)* | | | | | | | | | | | | |
| Jensen 2017 (11), Denmark | Copenhagen School Health Records Register (CSHRR) , men and women born in 1930 to 1972. Cancer-free in 1978. | 2,487 / 242,939 | Followed from 1978 or age 40 y to 2012 (mean 21.1 y). Age range at diagnosis 40-83 y. Cancer registry. | Measured height at age 13 y. | CC | Per z-score | 1.14 (1.09-1.19) | Age (as time axis), birth cohort, and sex. | * * * * | * * | * * * | 9 |
| | | 1,553 / 242,939 | | | RC | Per z-score | 1.03 (0.98-1.08) | | | | | |

Abbreviations: CC, colon cancer; CRC, colorectal cancer; F, female; M, male; RC, rectal cancer. NOS QS (NOS quality score): For selection, four stars could be awarded based on being: (1) representativeness of the exposed cohort; (2) selection of the non-exposed cohort; (3) ascertainment of exposure from a secure record, measured objectively, or a validated self-report method; and (4) demonstration that CRC was not present at the start of the study. For comparability, studies could score one star when they controlled for sex (if applicable). Another star was awarded when the study controlled for age/birth year, socio-economic status, race/ethnicity, adolescent smoking (status), adolescent physical activity, or adolescent alcohol intake. For outcome, 3 stars could be awarded: (1) outcome assessment through linkage with a cancer registry or verified self-report; (2) when the follow-up period was 10 years or more and the mean age of the population at diagnosis was at least 50 years (to assure sufficient cancer cases when follow-up started during early life); (3) follow-up rate ≥ 80% or outcome assessment through linkage with a cancer registry.

## Supplementary Table S5 Results from studies examining the association between young adult BMI and risk of colorectal cancer in adulthood

| | | | | | | | | | NOS QS | | | |
|---|---|---|---|---|---|---|---|---|---|---|---|---|
| Author, Year, Country | Study name (study design), Characteristics | Cases / Study size* | Length of follow-up, age at start and/or end follow-up or CRC diagnosis (range), case ascertainment | Exposure assessment, / age at exposure assessment | Subsite (sex) | Comparison | RR (95% CI) P-trend | Adjustment factors | Selection | Comparability | Outcome | |
| *Young adult BMI (from 18 until 25 years)* | | | | | | | | | | | | |
| Mariosa 2022 (19), Europe | European Prospective Investigation into Cancer and Nutrition (EPIC) (cohort), cancer-free men and women aged 40-69 y at study entry in 1992-1999. | 3,104 / 185,361 | Followed from study entry to 2008-2013 (max 21 y). Cancer registry or self-report verified by medical records. | Self-reported BMI age 18-20 y / 40-69 y | CRC | Per 5 $kg/m^2$ | 1.06 (0.99-1.13) | Age (as time-axis). Stratified by study centre and sex. | ** | ** | *** | 7 |
| Celind 2019 (10), Sweden | Gothenburg (cohort), men born in 1946 to 1961. | 257 / 37,663 | Followed from age 20 y to 2013 (mean 37.7 y). Cancer registry. | Measured BMI at age 17.5-22 y (age-adjusted to 20 y) | CC (M) | Per SD (2.5 $kg/m^2$) | 1.11 (0.98-1.25) | Birth year and country of birth. | **** | ** | *** | 9 |
| | | | | | RC (M) | Per SD (2.5 $kg/m^2$) | 0.88 (0.75-1.04) | | | | | |
| Liu 2019 (20), USA | Nurses' Health Study II (NHSII) (cohort), cancer-free women aged 25-42 at study entry in 1989. Women with inflammatory bowel were excluded. | 155 / 85,256 | From 1989 to 2011 (median 13.9 y). Diagnosis after age ≥50 y (CRC <50 y was censored). Self-report verified by medical records. | Self-reported BMI at early adulthood (age 18 y) / 25-42 y | CRC (F) | Per 5 $kg/m^2$ | 1.19 (0.94-1.49) | Height, family history of CRC, history of diabetes, endoscopy, smoking pack-years, physical activity, alcohol intake, aspirin use, NSAID, multivitamin, menopausal status, menopausal hormone use, and dietary intake. Stratified by age and study period. | ** | ** | *** | 7 |
| Pang 2018 (21), China | China Kadoorie Biobank (CKB) (cohort), cancer-free men and women aged 30-79 y at study entry in 2004-2008. | 3,024 / 509,568 | Followed until 2016 (10 y). Cancer registry. | Self-reported BMI age 25 y / 30-79 y (mean 52 y) | CRC | Per SD (2.6 $kg/m^2$) | 1.04 (1.00-1.08) | Education, smoking, alcohol, physical activity, fruits, vegetables, red meat, dairy products. Stratified by age, sex, and study area. | ** | ** | *** | 7 |
| Zheng 2018 (22), USA | Prostate, Lung, Colorectal, and Ovarian Cancer Screening trial (PLCO) (cohort), cancer-free men and women aged 49-78 y at study entry (mean 62.6 ± 5.3 y). | 2,031 / 139,229 | Median follow-up of 11.9 y. Mean age at diagnosis 69.8 ± 6.4 y. | Self-reported BMI at age 20 y / mean age 62.6 y | CRC | Per 5 $kg/m^2$ | 1.14 (1.05-1.24) | Randomization arm, sex, study center, race, family history of CRC, smoking status. | ** | ** | ** | 6 |

| | | | | | | | | | | | | |
|---|---|---|---|---|---|---|---|---|---|---|---|---|
| | | | Self-reported cancer verified by medical records. | | | | | | | | | |
| Kantor 2016 (23), Sweden | Swedish Military Conscription Register (cohort), men who underwent a compulsory conscription assessment for the Swedish military between 1969-1976 (born in 1952-1956). Men were excluded when they had inflammatory bowel disease or a history of CRC. | 885 / 239,658 | Followed from conscription examination to 2010 (mean 35 y).<br>Cancer registry. | Measured BMI at mean age 18.5 y. | CRC (M) | <18.5 vs 18.5-<25 kg/m$^2$ | 0.86 (0.68-1.08) | Age at conscription, erythrocyte volume fraction, household crowding health status, blood pressure, muscular strength, physical working capacity, cognitive functioning, and erythrocyte sedimentation rate. | * * * * | * * | * * * | 9 |
| | | | | | | 25-27.5 vs 18.5-<25 kg/m$^2$ | 1.15 (0.85-1.55) | | | | | |
| | | | | | | 27.5-<30 vs 18.5-<25 kg/m$^2$ | 2.08 (1.40-3.07) | | | | | |
| | | | | | | >30 vs 18.5-<25 kg/m$^2$ | 2.38 (1.51-3.76) | | | | | |
| Zhang 2015 (14), USA | Nurses' Health Study (NHS) (cohort), women aged 30-55 y in 1976. Cancer-free in 1988 (mean age 55 y). Women were excluded when they had ulcerative colitis. | 1,292 / 75,238 | Followed from 1988 to 2010 (22 y).<br>Self-reported cancer verified by medical records. | Self-reported BMI 18 y / 40-65 y (mean 55 y) | CRC (F) | 18.5-<23.0 vs 15-<18.5 kg/m$^2$ | 1.06 (0.88-1.28) | Age, height, smoking before age 30, family history of CRC, history of sigmoidoscopy /endoscopy, current physical activity, aspirin use, processed meat, and postmenopausal hormone use. | * * | * * | * * * | 7 |
| | | | | | | 23.0-<25 vs 15-<18.5 kg/m$^2$ | 1.21 (0.95-1.53) | | | | | |
| | | | | | | 25.0-<27.5 vs 15-<18.5 kg/m$^2$ | 1.33 (1.01-1.74) | | | | | |
| | | | | | | 27.5-<30 vs 15-<18.5 kg/m$^2$ | 1.43 (0.95-1.53) | | | | | |
| | | | | | | ≥30-<45 vs 15-<18.5 kg/m$^2$ | 1.71 (1.16-2.51) | | | | | |
| | Health Professionals Follow-Up Study HPFS (cohort), men aged 40-75 y in 1986. Cancer-free in 1988 (mean age 56 y). Men were excluded when they had ulcerative colitis. | 808 / 34,533 | | Self-reported BMI 21 y / 40-75 y (mean 56 y) | CRC (M) | 18.5-<23.0 vs 15-<18.5 kg/m$^2$ | 1.10 (0.84-1.46) | | * * | * * | * * * | 7 |
| | | | | | | 23.0-<25 vs 15-<18.5 kg/m$^2$ | 1.14 (0.84-1.53) | | | | | |
| | | | | | | 25.0-<27.5 vs 15-<18.5 kg/m$^2$ | 1.09 (0.79-1.51) | | | | | |
| | | | | | | 27.5-<30 vs 15-<18.5 kg/m$^2$ | 1.13 (0.76-1.69) | | | | | |
| | | | | | | ≥30-<45 vs 15-<18.5 kg/m$^2$ | 1.11 (0.70-1.76) | | | | | |

| | | | | | | | | | | | | |
|---|---|---|---|---|---|---|---|---|---|---|---|---|
| Han 2014 (24), USA | Atherosclerosis Risk in Communities Study (ARIC) (cohort), cancer-free men and women aged 45-64 y at study entry in 1987-1989. | 151 / 6,332 | Followed from 1987 until 2006 (max 19 y). Cancer registry supplemented by hospital records. | Self-reported BMI at age 25 y / 45-64 y | CRC (M) | Per 5 kg/m$^2$ | 1.14 (0.91-1.44) | Race-center, age, education, height, smoking status at age 25, age at menarche, smoking at study entry, alcohol consumption at study entry, physical activity at study entry, menopause status, and age at menopause. | * * | * * | * * * | 7 |
| | | 147 / 7,569 | | | CRC (F) | Per 5 kg/m$^2$ | 1.01 (0.80-1.26) | | | | | |
| Li 2013 (25), China | Shanghai Women's Health Study (SWHS) (cohort), cancer-free women aged 40-70 y at study entry in 1997-2000. | 622 / 62,236 | From study entry until 2009 (mean 11.0 y). Cancer registry and verified self-report. | Self-reported BMI at age 20 y / 40-70 y | CRC (F) | 17.54-18.72 vs <17.54 kg/m$^2$ | 1.03 (0.77-1.39) | Age (as time-axis), education, income, smoking, alcohol consumption, tea consumption, physical activity, family history of CRC, menopausal status, and intakes of energy, red meat, fruit and vegetables. | * * | * * | * * * | 7 |
| | | | | | | 18.73-19.99 vs <17.54 kg/m$^2$ | 1.17 (0.88-1.55) | | | | | |
| | | | | | | 20.00-21.62 vs <17.54 kg/m$^2$ | 0.99 (0.73-1.33) | | | | | |
| | | | | | | ≥21.63 vs <17.54 kg/m$^2$ | 1.08 (0.81-1.44) | | | | | |
| | Shanghai Men's Health Study SMHS (cohort), cancer-free men aged 40-74 y at study entry in 2002-2006. | 313 / 52,894 | From study entry until 2009 (mean 5.5 y). Cancer registry and verified self-report. | Self-reported BMI at age 20 y / 40-74 y | CRC (M) | 17.93-19.02 vs <17.93 kg/m$^2$ | 0.96 (0.62-1.48) | | * * | * * | * * | 6 |
| | | | | | | 19.03-19.89 vs <17.93 kg/m$^2$ | 1.08 (0.71-1.64) | | | | | |
| | | | | | | 19.90-21.00 vs <17.93 kg/m$^2$ | 1.19 (0.79-1.79) | | | | | |
| | | | | | | ≥21.01 vs <17.93 kg/m$^2$ | 1.21 (0.81-1.79) | | | | | |
| Renehan 2012 (26), USA | NIH-AARP diet and health (cohort), cancer-free men and women (39%) aged 50-71 y (mean 63 y) at study entry in 1995. | 2804 / 168,294 | Followed from 1996 to 2006 (max 10 y). Cancer registry. | Self-reported BMI age 18 y / 51-72 y | CRC (M) | Per 5 kg/m$^2$ | 1.08 (0.99-1.17) | Age, race/ethnicity, education, physical activity, smoking, alcohol consumption, and menopausal hormone therapy. | * * | * * | * * * | 7 |
| | | 1240 / 105,385 | | | CRC (F) | Per 5 kg/m$^2$ | 1.07 (0.93-1.22) | | | | | |
| Hughes 2011 (27), The Netherlands | Netherlands Cohort Study (NLCS) (case-cohort), cancer-free men and women (57%) aged 55-69 y at study entry in 1986. | 1,211 / 1,365 | Follow-up of 18,595 person-years (mean 13.6 y). Cancer registry. | Self-reported BMI at age 20 y / 55-69 y | CRC (M) | Per 5 kg/m$^2$ | 1.15 (0.94-1.37) | Age, energy intake, physical activity, education, family history of CRC, alcohol consumption, and smoking. | * * | * * | * * * | 7 |
| | | 1,106 / 1,832 | Follow-up of 27,195 person-years (mean 14.8 y). Cancer registry. | | CRC (F) | Per 5 kg/m$^2$ | 1.04 (0.90-1.19) | | | | | |

| | | | | | | | | | | | | |
|---|---|---|---|---|---|---|---|---|---|---|---|---|
| Win 2011 (28), Australia, New Zealand, USA, Canada | Colon Cancer Family Registry (CFR) (cohort), men and women, study entry between 1997-2007. Confirmed non-carriers of an MMR gene mutation. | 36 / 1,219 | Followed from birth (mean 52 y). Mean age at diagnosis 56.8 (14.8) y. Verified self-report. | Self-reported BMI at age 20 y / at study entry | CRC | Per 5 kg/m² | 1.64 (1.02-2.64) | Age (as time axis), sex, country, smoking, alcohol drinking. | * * | * * | * * | 6 |
| | Confirmed carriers of an MMR gene mutation. | 659 / 1,324 | Followed from birth (mean 44 y). Mean age at diagnosis 44.0 (11.1) y. Verified self-report. | | | Per 5 kg/m² | 1.30 (1.08-1.58) | Further adjusted for specific MMR gene mutation. | * * | * * | * | 5 |
| Bassett 2010 (29), Australia | Melbourne Collaborative Cohort Study (MCCS) (cohort), CRC-free men and women aged 40-69 y at study entry in 1990-1994. | 569 / 41,514 | Followed from 1990/94 until 2007 (mean 14 y). Mean age at diagnosis 68 y (range 41-85 y). Cancer registry. | Self-reported BMI at age 18-21 y (BMI at 18)/ 40-69 y | CC (M) | Per 5 kg/m² | 1.05 (0.80-1.37 | Age (as time axis), country of birth, education, processed and fresh meat consumption, fruit and vegetable consumption, fat intake, daily energy intake, smoking status, and alcohol consumption. | * * | * * | * * * | 7 |
| | | | | | CC (F) | Per 5 kg/m² | 1.19 (0.93-1.52) | | | | | |
| Oxentenko 2010 (30), USA | Iowa Women's Health Study (IWHS) (cohort), cancer-free women aged 55-69 y at study entry in 1986. | 1464 / 36,941 | Followed from 1986 to 2005 (max 19 y). Cancer registry. | Self-reported BMI age 18 y / 55-69 y | CRC (F) | <18.5 vs 18.5-24.9 kg/m² | 0.93 (0.78-1.12) | Age (as time axis), age at menopause, exogenous estrogen use, oral contraceptive use, smoking status, cigarette pack-years, physical activity, diabetes mellitus, intake of total energy, total fat, red meat, fruits and vegetables, calcium, folate, vitamin E, and alcohol. | * * | * * | * * * | 7 |
| | | | | | | 25.0-29.9 vs 18.5-24.9 kg/m² | 1.17 (0.98-1.40) | | | | | |
| | | | | | | 30.0-34.9 vs 18.5-24.9 kg/m² | 0.81 (0.51-1.27) | | | | | |
| | | | | | | ≥35.0 vs 18.5-24.9 kg/m² | 0.43 (0.11-1.68) | | | | | |
| Lee 1992 (31), USA | Harvard Alumni Health Study (cohort), cancer-free men at college entry in 1916-1950. | 266 / 15,361 | Followed from 1962/1966 (mean age 47 y) to 1988 (max 26 y). Median age at diagnosis 55 y. Self-reported cancer. | Measured BMI at college entry / ~18 y | CC (M) | Per 1 kg/m² | 1.05 (1.00-1.10) | Age, physical activity, and parental history of cancer. | * * * | * * | * * | 7 |

Abbreviations: CC, colon cancer; CRC, colorectal cancer; F, female; M, male; RC, rectal cancer. NOS QS (NOS quality score): For selection, four stars could be awarded based on being: (1) representativeness of the exposed cohort; (2) selection of the non-exposed cohort; (3) ascertainment of exposure from a secure record, measured objectively, or a validated self-report method; and (4) demonstration that CRC was not present at the start of the study. For comparability, studies could score one star when they controlled for sex (if applicable). Another star was awarded when the study controlled for age/birth year, socio-economic status, race/ethnicity, young adult smoking (status), young adult physical activity, or young adult alcohol intake. For outcome, 3 stars could be awarded: (1) outcome assessment through linkage with a cancer registry or verified self-report; (2) when the follow-up period was 10 years or more and the mean age of the population at diagnosis was at least 50 years (to assure sufficient cancer cases when follow-up started during early life); (3) follow-up rate ≥ 80% or outcome assessment through linkage with a cancer registry.

## Supplementary Table S6 Results from studies examining the association between change in early life BMI and risk of colorectal cancer in adulthood

| | | | | | | | | | NOS QS | | | |
|---|---|---|---|---|---|---|---|---|---|---|---|---|
| Author, Year, Country | Study name (study design), Characteristics | Cases / Study size* | Length of follow-up, age at start and/or end follow-up or CRC diagnosis (range), case ascertainment | Exposure assessment, / age at exposure assessment | Subsite (sex) | Comparison | RR (95% CI) P-trend | Adjustment factors | Selection | Comparability | Outcome | |
| *Change in early-life BMI* | | | | | | | | | | | | |
| Celind 2019 (10), Sweden | Gothenburg (cohort), men born in 1946 to 1961. | 257 / 37,663 | Followed from age 20 y to 2013 (mean 37.7 y). Cancer registry. | Measured BMI between 6.5-9.5 y (age-adjusted to 8 y) and at age 17.5-22 y (age-adjusted to 20 y) | CC (M) | Per SD increase in BMI | 1.02 (0.90-1.16) | Birth year and country of birth. | * * * * | * * | * * * | 9 |
| | | 159 / 37,663 | | | RC (M) | Per SD change in BMI | 0.88 (0.74-1.04) | | | | | |
| Jensen 2017 (11), Denmark | Copenhagen School Health Records Register (CSHRR) (cohort), men and women born in 1930 to 1972. Cancer-free in 1978. | 2,487 / 242,939 | Followed from 1978 or 40 y to 2012 (mean 21 y). Age range at diagnosis 40-83 y. Cancer registry. | Measured BMI at age 7 y at age 13 y. | CC (M) | Increasing BMI more than average vs average increase | 1.08 (1.03-1.14) | Stratified by birth cohort. | * * * * | * * | * * * | 9 |
| | | | | | CC (F) | | 1.03 (0.98-1.08) | | | | | |
| | | 1,553 / 242,939 | | | RC (M) | | 1.03 (0.97-1.09) | | | | | |
| | | | | | RC (F) | | 0.99 (0.92-1.06) | | | | | |

Abbreviations: CC, colon cancer; F, female; M, male; RC, rectal cancer. NOS QS (NOS quality score): For selection, four stars could be awarded based on being: (1) representativeness of the exposed cohort; (2) selection of the non-exposed cohort; (3) ascertainment of exposure from a secure record, measured objectively, or a validated self-report method; and (4) demonstration that CRC was not present at the start of the study. For comparability, studies could score one star when they controlled for sex (if applicable). Another star was awarded when the study controlled for age/birth year, socio-economic status, race/ethnicity, early-life smoking (status), early-life physical activity, or early-life alcohol intake. For outcome, 3 stars could be awarded: (1) outcome assessment through linkage with a cancer registry or verified self-report; (2) when the follow-up period was 10 years or more and the mean age of the population at diagnosis was at least 50 years (to assure sufficient cancer cases when follow-up started during early life); (3) follow-up rate ≥ 80% or outcome assessment through linkage with a cancer registry.

## Supplementary Table S7 Grading criteria for evidence on diet, nutrition, physical activity and cancer incidence

| Evidence grades | | GRADING CRITERIA FOR EVIDENCE ON DIET, NUTRITION, PHYSICAL ACTIVITY AND CANCER INCIDENCE | Het | PB | Mec |
|---|---|---|---|---|---|
| Strong evidence | Convincing | Evidence from more than one good quality study type, including at least two independent cohort studies (acceptable study designs listed below)* | No | No | Required |
| | Probable | Evidence from at least two good quality independent cohort studies | No | No | Required |
| Limited evidence | Limited suggestive | Evidence from at least two independent cohort studies | Yes | Yes | Required |
| | Limited – no conclusion | Any of the following reasons:<br>- Too few studies available<br>- Inconsistency of direction of effect<br>- Magnitude of effect unlikely to affect cancer risk<br>- Poor quality of studies (for example, lack of adjustment for known confounders) | - | - | - |
| Strong evidence | Substantial effect on risk unlikely | Evidence of the absence of an effect. All of the following generally required:<br>- Evidence from more than one good quality study type (acceptable study designs listed below)*<br>- Evidence from at least two independent cohort studies<br>- Summary estimate of effect close to 1.0 for comparison of high- versus low-exposure categories | No | No | Absence |

**Het:** Substantial unexplained heterogeneity or some unexplained heterogeneity
**PB:** Publication bias
**Mec:** Strong and plausible mechanistic evidence is required, desirable but not required, not required, or absent
*RCTs, longitudinal, observational, or pooled analyses of individual data of these studies. Good-quality studies exclude with confidence the possibility that the observed association results from random or systematic error, including *confounding*, *measurement error* and *selection bias*.

**Special upgrading factors:**
- Presence of a plausible biological gradient ('dose response') in the association. Such a gradient need not be linear or even in the same direction across the different levels of exposure, so long as this can be explained plausibly. This is also a requirement for the convincing (strong evidence) grade.
- A particularly large summary effect size (an odds ratio or relative risk of 2.0 or more, depending on the unit of exposure), after appropriate control for confounders.
- Consideration of precision.
- Evidence from randomised trials in humans.
- Evidence from appropriately controlled experiments demonstrating one or more plausible and specific mechanisms operating in humans.
- Robust and reproducible evidence from experimental studies in appropriate animal models showing that typical human exposures can lead to relevant cancer outcomes.
- Supportive evidence from Mendelian randomization studies.

**Supplementary Table S8. Studies excluded and reason of exclusion from the potentially eligible studies after further inspection**

| **Reason of exclusion** | **Number of studies** | **Reference** |
|---|---|---|
| 17 publications excluded after further inspection | | |
| Overlapping study population | 8 | (33-40) |
| Early-onset colorectal cancer | 3 | (41-43) |
| Case-control study | 5 | (44-48) |
| Missing information (exposure category values) | 1 | (32) |

## Supplementary Table S9 Results from Mendelian randomization (MR) studies examining the association between genetically predicted anthropometry in early life and risk of colorectal cancer in adulthood

| Author, year | Exposure | Exposure population source and ancestry | No. IVs / $R^2$ | Outcome | Outcome population source and ancestry | MR design, main analysis method | Comparison | Main analysis RR (95% CI) | Sensitivity analyses performed |
|---|---|---|---|---|---|---|---|---|---|
| *Intra-uterine life (from conception until birth, including measurements at birth) – Birth weight* | | | | | | | | | |
| Chen 2022 (49) | Birth weight | GWAS / Horikoshi et al, 2013<br>European<br>Excluding non-singletons and premature births. | 60 | CRC (M)<br>Cases: 5,046<br>Controls: 439,280 | MRC-IEU<br>European | Two-sample,<br>IVW random | Not specified | 1.00 (0.99-1.00) | MR Egger<br>Weighted median<br>MR-PRESSO |
| Cornish 2020 (50) | Birth weight | GWAS UK Biobank<br>European | 93<br>2.5% | CRC<br>Cases: 26,397<br>Controls: 41,481 | GWAS / Law et al, 2019<br>(excluding UK Biobank)<br>European | Two-sample,<br>Maximum likelihood random | 1 SD | 1.10 (0.92-1.31) | MR Egger<br>Weighted median |
| Gao 2016 (51) | Birth weight | GWAS / Horikoshi et al, 2013<br>European | 7 | CRC<br>Cases: 5,00<br>Controls: 4,831 | GAME-ON / CORECT<br>European | Two-sample,<br>IVW fixed | 1 SD | 0.69 (0.44-1.10) | MR Egger |
| Jarvis 2016 (52) | Birth weight<br>N=69,308 | GWAS / Horikoshi et al, 2013<br>European | 7 | CRC<br>Cases: 9,254<br>Controls: 18,386 | GWAS / Orlando et al, 2016<br>European | Two-sample,<br>Meta-analysis of two-sample IVW MR estimates | Per kg | 1.22 (0.89-1.67) | MR Egger |
| *Childhood BMI (from 2 until <10 years)* | | | | | | | | | |
| Fang 2021 (53) | Childhood BMI | ECG<br>European | 15 | CRC<br>Cases: 6,375<br>Controls: 401,350 | UK Biobank<br>European | Two-sample<br>IVW random | 1 SD | 1.11 (0.93-1.32) | MR Egger<br>Weighted median |
| Gao 2016 (51) | Childhood BMI | GWAS / Felix et al, 2016<br>European | 15 | CRC<br>Cases: 5,100<br>Controls: 4,831 | GAME-ON / CORECT<br>European | Two-sample,<br>IVW fixed | 1 SD | 1.20 (0.90-1.59) | MR Egger |
| Jarvis 2016 (52) | Childhood obesity<br>N=9,850 | GWAS / Wheeler et al, 2013<br>European | 9 | CRC<br>Cases: 9254<br>Controls: 18,386 | GWAS / Orlando et al, 2016<br>European | Two-sample, Meta-analysis of two-sample IVW MR estimates | Not specified | 1.07 (1.01-1.13) | MR Egger |
| *Adolescent body fatness (from 10 until ~19 years)* | | | | | | | | | |
| Papadimitriou 2023 (54) | Early life body size (at age 10)<br>N=453,169 | GWAS UK Biobank | 305<br>4.5% | CRC<br>Cases: 52,775<br>Controls: 45,940 | CORECT, CCFR, GECCO<br>(not including UK Biobank) | Two-sample,<br>IVW fixed | Per category increase<br>(thinner, plumper, about average) | 1.12 (0.98-1.27) | MR Egger<br>Weighted mean |
| | | | | 27,817 CC | | | | 1.16 (1.00-1.35) | |
| | | | | 12,360 proximal CC | | | | 1.11 (0.93-1.32) | |
| | | | | 14,016 distal CC | | | | 1.25 (1.04-1.51) | |
| | | | | 13,713 RC | | | | 1.14 (0.93-1.36) | |

| | | | | | | | | | |
|---|---|---|---|---|---|---|---|---|---|
| Mariosa 2022 (19) | Early life body size (at age 10) N=453,169 | GWAS UK Biobank | 195 4.1% | CRC Cases: 5,100 Controls: 4,831 | GWAS / Schmit et al, 2019 | Two-sample, IVW random | Per category change (thinner, plumper, about average) | 1.25 (0.89-1.77) | Multivariable MR |

CC, colon cancer; CI, confidence interval ; CRC, colorectal cancer; GWAS, Genome-Wide Association Study; IIVs, instrumental variables; IVW, inverse-variance weighted method;; M, male; MR-PRESSO, Mendelian Randomization Pleiotropy Residual Sum and Outlier; RC, rectal cancer; RR, relative risk; SD, standard deviation.

## Supplementary Figure S1: Flowchart of study selection process

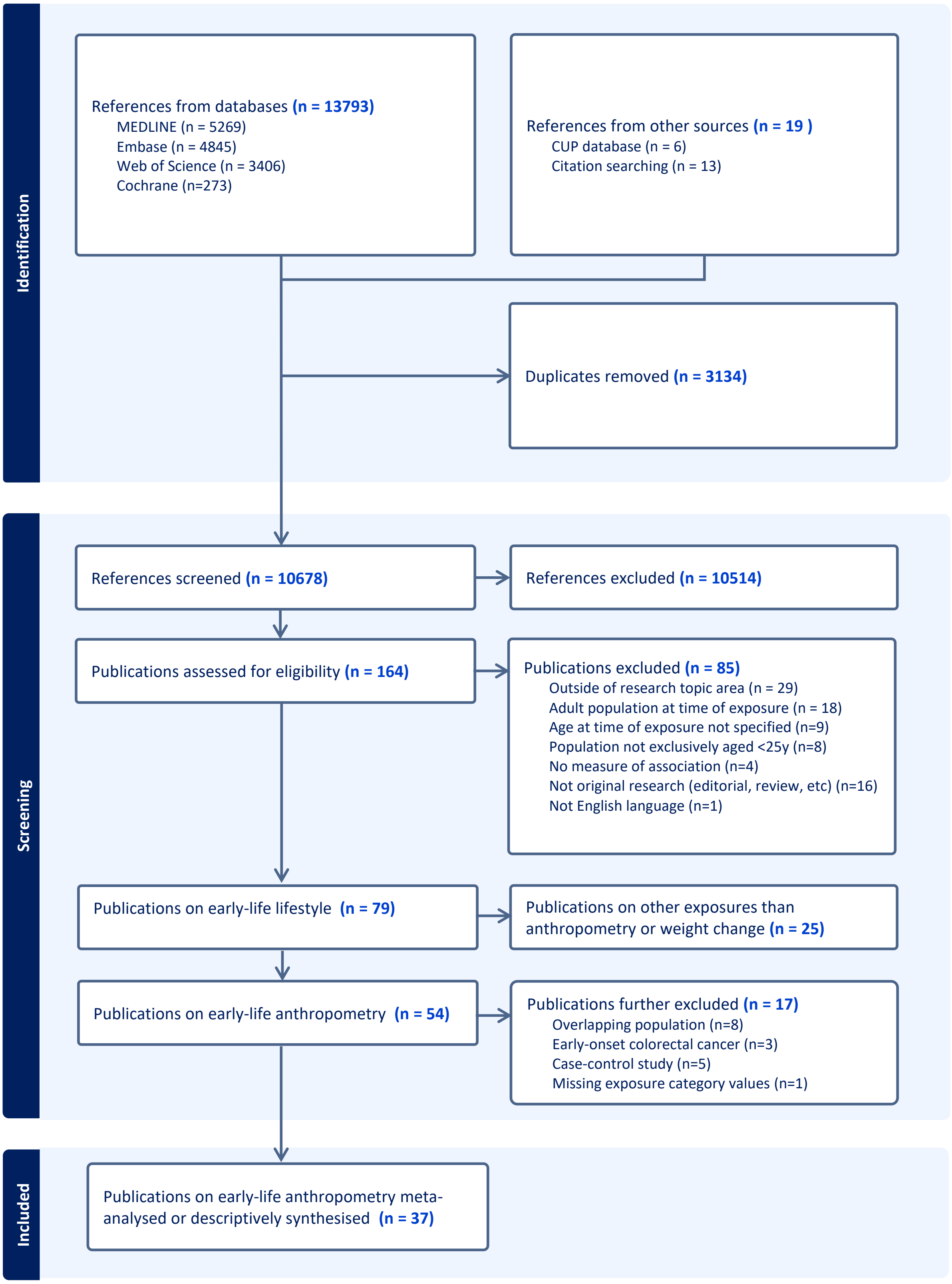


**Figure S1. Flowchart of study selection process**

**Supplementary Figure S2: Funnel plot of studies included in the linear dose-response meta-analysis of young adult BMI and colorectal cancer risk in adulthood**

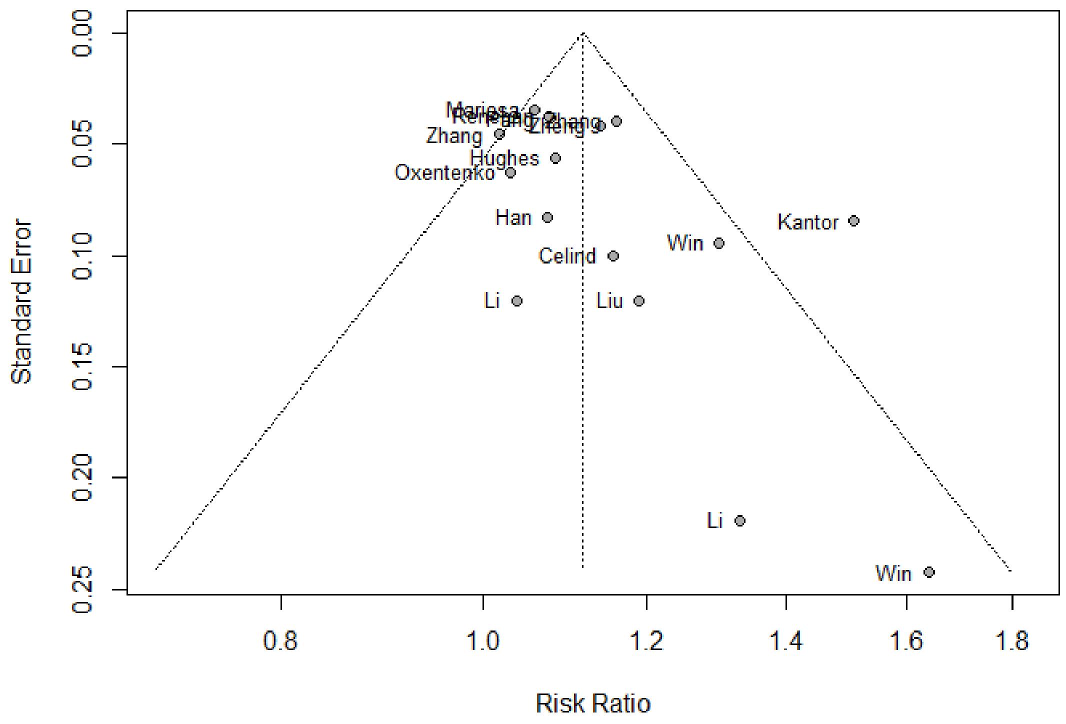


P Egger’s test = 0.06

**Supplementary Figure S2. Funnel plot young adult BMI.** Each point represents the relative risk (RR) estimate (x-axis) against its standard error as measure of study size (y-axis). Dashed vertical line is the summary RR from the random effect meta-analysis.

**Supplementary Figure S3: Meta-analyses young adult adiposity stratified by tumour subsites**

A

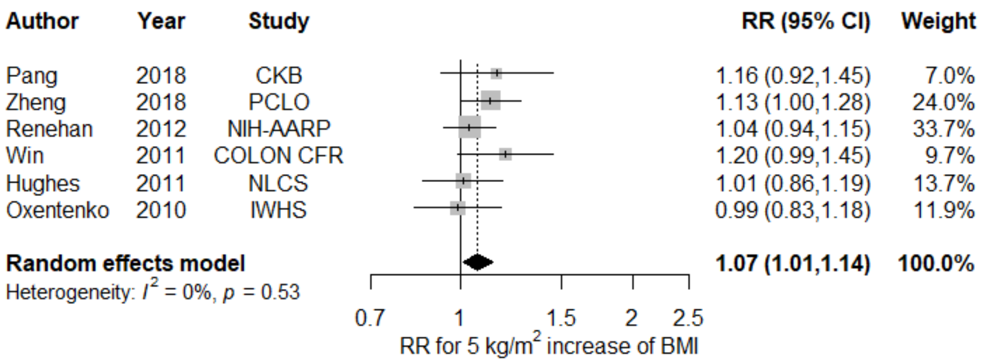


B

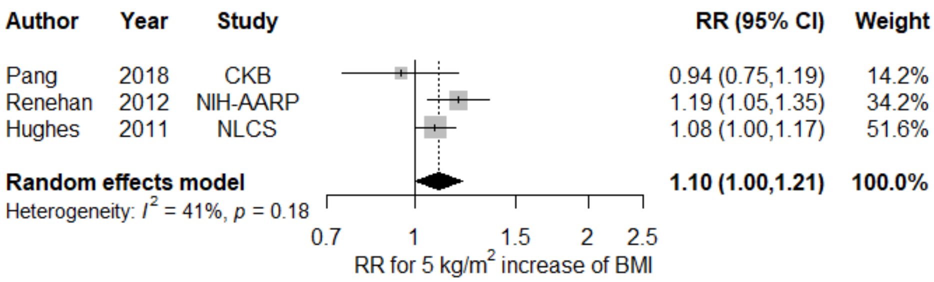


**Supplementary Figure S3. Linear and nonlinear dose-response relationships between young adult body fatness and proximal or distal colon cancer risk in adulthood.** This figure shows the results from **A)** linear dose-response relationships between BMI and proximal colon cancer risk*;* **B)** linear dose-response relationships between BMI and distal colon cancer. The black diamond represents the summary relative risk (RR) estimate, with its width indicating the 95% confidence interval (CI). The squares and horizontal lines show study-specific RRs and their 95% CIs. The area of each grey square reflects the study's weight in the meta-analysis. The increment unit is 5 kg/m$^2$.

**Supplementary Figure S4: Leave-one-out analyses of birthweight**

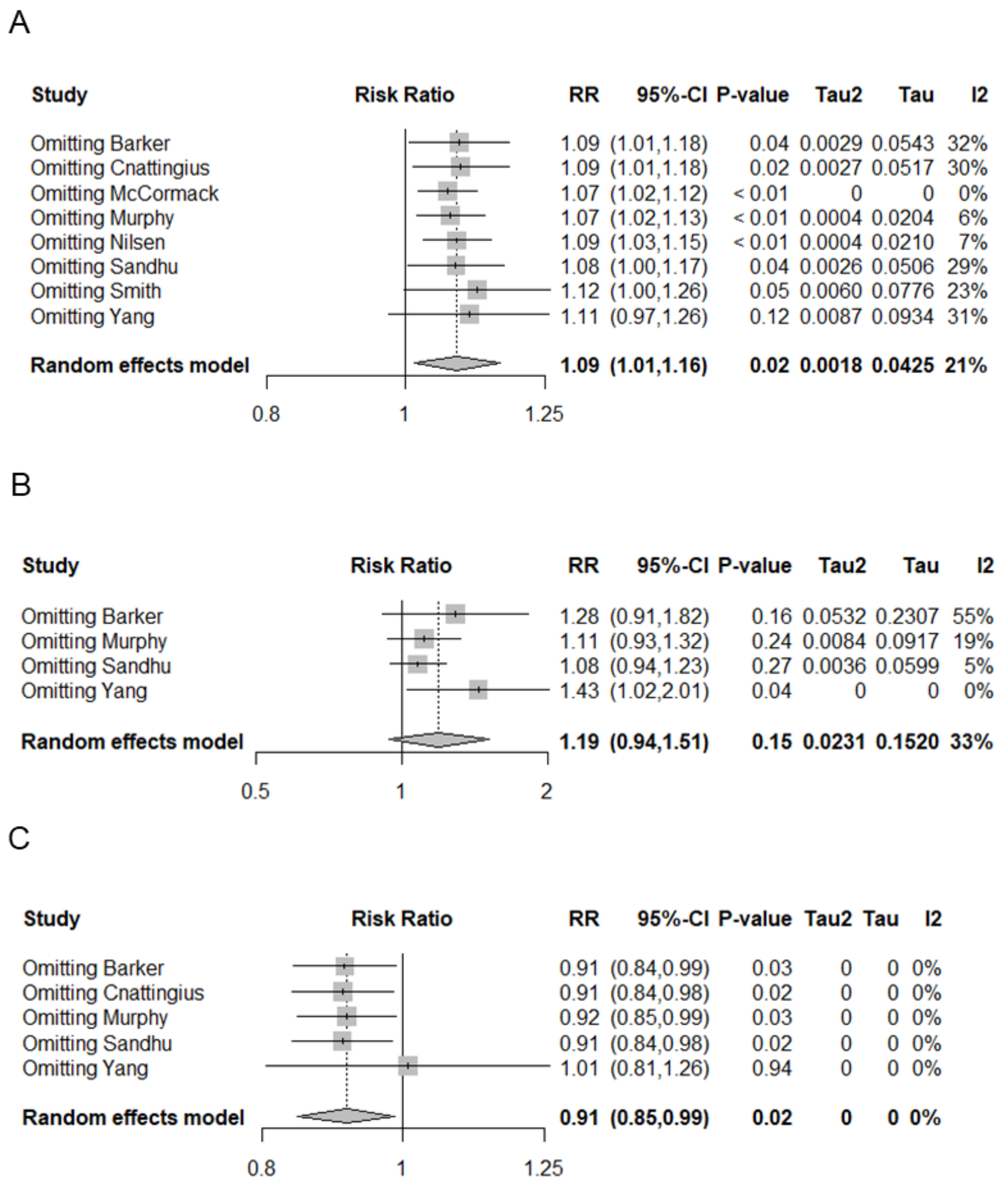


**Supplementary Figure S4. Leave-one-out sensitivity analyses birthweight and colorectal cancer risk in adulthood**. This figure shows the results for **A)** linear dose-response analysis; **B)** comparison of high (>4,000g) vs. normal (2,500-4,000) birthweight **C)** comparison of low (<2,500g) vs. normal (2,500-4,000g) birthweight. The diamond represents the summary relative risk (RR) estimate, with its width indicating the 95% confidence interval (CI). The squares and horizontal lines show RRs and their 95% CIs after omitting one study. The increment unit is 1,000g.

## References


1. Murphy CC, Cirillo PM, Krigbaum NY, Singal AG, Lee M, Zaki T, Burstein E and Cohn BA: Maternal obesity, pregnancy weight gain, and birth weight and risk of colorectal cancer. Gut 71: 1332-1339, 2022.
2. Smith NR, Jensen BW, Zimmermann E, Gamborg M, Sorensen TI and Baker JL: Associations between birth weight and colon and rectal cancer risk in adulthood. Cancer Epidemiol 42: 181-185, 2016.
3. Spracklen CN, Wallace RB, Sealy-Jefferson S, Robinson JG, Freudenheim JL, Wellons MF, Saftlas AF, Snetselaar LG, Manson JE, Hou L, Qi L, Chlebowski RT and Ryckman KK: Birth weight and subsequent risk of cancer. Cancer Epidemiol 38: 538-543, 2014.
4. Yang TO, Reeves GK, Green J, Beral V, Cairns BJ and Million Women Study C: Birth weight and adult cancer incidence: large prospective study and meta-analysis. Ann Oncol 25: 1836-1843, 2014.
5. Barker DJ, Osmond C, Thornburg KL, Kajantie E and Eriksson JG: The shape of the placental surface at birth and colorectal cancer in later life. Am J Hum Biol 25: 566-568, 2013.
6. Cnattingius S, Lundberg F and Iliadou A: Birth characteristics and risk of colorectal cancer: a study among Swedish twins. Br J Cancer 100: 803-806, 2009.
7. McCormack VA, dos Santos Silva I, Koupil I, Leon DA and Lithell HO: Birth characteristics and adult cancer incidence: Swedish cohort of over 11,000 men and women. Int J Cancer 115: 611-617, 2005.
8. Nilsen TI, Romundstad PR, Troisi R, Potischman N and Vatten LJ: Birth size and colorectal cancer risk: a prospective population based study. Gut 54: 1728-1732, 2005.
9. Sandhu MS, Luben R, Day NE and Khaw KT: Self-reported birth weight and subsequent risk of colorectal cancer. Cancer Epidemiol Biomarkers Prev 11: 935-938, 2002.
10. Celind J, Ohlsson C, Bygdell M, Nethander M and Kindblom JM: Childhood Body Mass Index Is Associated with Risk of Adult Colon Cancer in Men: An Association Modulated by Pubertal Change in Body Mass Index. Cancer Epidemiol Biomarkers Prev 28: 974-979, 2019.
11. Jensen BW, Gamborg M, Gogenur I, Renehan AG, Sorensen TIA and Baker JL: Childhood body mass index and height in relation to site-specific risks of colorectal cancers in adult life. Eur J Epidemiol 32: 1097-1106, 2017.
12. Jeffreys M, Smith GD, Martin RM, Frankel S and Gunnell D: Childhood body mass index and later cancer risk: a 50-year follow-up of the Boyd Orr study. Int J Cancer 112: 348-351, 2004.
13. Li J, Eriksson M, He W, Hall P and Czene K: Associations between childhood body size and seventeen adverse outcomes: analysis of 65,057 European women. Sci Rep 7: 16917, 2017.
14. Zhang X, Wu K, Giovannucci EL, Ma J, Colditz GA, Fuchs CS, Willett WC, Stampfer MJ, Nimptsch K, Ogino S and Wei EK: Early life body fatness and risk of colorectal cancer in u.s. Women and men-results from two large cohort studies. Cancer Epidemiol Biomarkers Prev 24: 690-697, 2015.
15. Whitley E, Martin RM, Smith GD, Holly JM and Gunnell D: Childhood stature and adult cancer risk: the Boyd Orr cohort. Cancer Causes Control 20: 243-251, 2009.
16. Furer A, Afek A, Sommer A, Keinan-Boker L, Derazne E, Levi Z, Tzur D, Tiosano S, Shina A, Glick Y, Kark JD, Tirosh A and Twig G: Adolescent obesity and midlife cancer risk: a population-based cohort study of 2.3 million adolescents in Israel. Lancet Diabetes Endocrinol 8: 216-225, 2020.
17. Batty GD, Calvin CM, Brett CE, Cukic I and Deary IJ: Childhood body weight in relation to morbidity from cardiovascular disease and cancer in older adulthood: 67-year follow-up of participants in the 1947 Scottish Mental Survey. Am J Epidemiol 182: 775-780, 2015.
18. Must A, Jacques PF, Dallal GE, Bajema CJ and Dietz WH: Long-term morbidity and mortality of overweight adolescents. A follow-up of the Harvard Growth Study of 1922 to 1935. N Engl J Med 327: 1350-1355, 1992.

19. Mariosa D, Smith-Byrne K, Richardson TG, Ferrari P, Gunter MJ, Papadimitriou N, Murphy N, Christakoudi S, Tsilidis KK, Riboli E, Muller D, Purdue MP, Chanock SJ, Hung RJ, Amos CI, O'Mara TA, Amiano P, Pasanisi F, Rodriguez-Barranco M, Krogh V, Tjonneland A, Halkjaer J, Perez-Cornago A, Chirlaque MD, Skeie G, Rylander C, Borch KB, Aune D, Heath AK, Ward HA, Schulze M, Bonet C, Weiderpass E, Davey Smith G, Brennan P and Johansson M: Body Size at Different Ages and Risk of 6 Cancers: A Mendelian Randomization and Prospective Cohort Study. J Natl Cancer Inst 114: 1296-1300, 2022.
20. Liu PH, Wu K, Ng K, Zauber AG, Nguyen LH, Song M, He X, Fuchs CS, Ogino S, Willett WC, Chan AT, Giovannucci EL and Cao Y: Association of Obesity With Risk of Early-Onset Colorectal Cancer Among Women. JAMA Oncol 5: 37-44, 2019.
21. Pang Y, Kartsonaki C, Guo Y, Chen Y, Yang L, Bian Z, Bragg F, Millwood IY, Mao E, Li Y, Shi L, Chen J, Li L, Holmes MV and Chen Z: Adiposity and risks of colorectal and small intestine cancer in Chinese adults: a prospective study of 0.5 million people. Br J Cancer 119: 248-250, 2018.
22. Zheng R, Du M, Zhang B, Xin J, Chu H, Ni M, Zhang Z, Gu D and Wang M: Body mass index (BMI) trajectories and risk of colorectal cancer in the PLCO cohort. Br J Cancer 119: 130-132, 2018.
23. Kantor ED, Udumyan R, Signorello LB, Giovannucci EL, Montgomery S and Fall K: Adolescent body mass index and erythrocyte sedimentation rate in relation to colorectal cancer risk. Gut 65: 1289-1295, 2016.
24. Han X, Stevens J, Truesdale KP, Bradshaw PT, Kucharska-Newton A, Prizment AE, Platz EA and Joshu CE: Body mass index at early adulthood, subsequent weight change and cancer incidence and mortality. Int J Cancer 135: 2900-2909, 2014.
25. Li H, Yang G, Xiang YB, Zhang X, Zheng W, Gao YT and Shu XO: Body weight, fat distribution and colorectal cancer risk: a report from cohort studies of 134255 Chinese men and women. Int J Obes (Lond) 37: 783-789, 2013.
26. Renehan AG, Flood A, Adams KF, Olden M, Hollenbeck AR, Cross AJ and Leitzmann MF: Body mass index at different adult ages, weight change, and colorectal cancer risk in the National Institutes of Health-AARP Cohort. Am J Epidemiol 176: 1130-1140, 2012.
27. Hughes LA, Simons CC, van den Brandt PA, Goldbohm RA, van Engeland M and Weijenberg MP: Body size and colorectal cancer risk after 16.3 years of follow-up: an analysis from the Netherlands Cohort Study. Am J Epidemiol 174: 1127-1139, 2011.
28. Win AK, Dowty JG, English DR, Campbell PT, Young JP, Winship I, Macrae FA, Lipton L, Parry S, Young GP, Buchanan DD, Martinez ME, Jacobs ET, Ahnen DJ, Haile RW, Casey G, Baron JA, Lindor NM, Thibodeau SN, Newcomb PA, Potter JD, Le Marchand L, Gallinger S, Hopper JL and Jenkins MA: Body mass index in early adulthood and colorectal cancer risk for carriers and non-carriers of germline mutations in DNA mismatch repair genes. Br J Cancer 105: 162-169, 2011.
29. Bassett JK, Severi G, English DR, Baglietto L, Krishnan K, Hopper JL and Giles GG: Body size, weight change, and risk of colon cancer. Cancer Epidemiol Biomarkers Prev 19: 2978-2986, 2010.
30. Oxentenko AS, Bardia A, Vierkant RA, Wang AH, Anderson KE, Campbell PT, Sellers TA, Folsom AR, Cerhan JR and Limburg PJ: Body size and incident colorectal cancer: a prospective study of older women. Cancer Prev Res (Phila) 3: 1608-1620, 2010.
31. Lee IM and Paffenbarger RS, Jr.: Quetelet's index and risk of colon cancer in college alumni. J Natl Cancer Inst 84: 1326-1331, 1992.
32. Wu AH, Paganini-Hill A, Ross RK and Henderson BE: Alcohol, physical activity and other risk factors for colorectal cancer: a prospective study. Br J Cancer 55: 687-694, 1987.
33. Ahlgren M, Wohlfahrt J, Olsen LW, Sorensen TI and Melbye M: Birth weight and risk of cancer. Cancer 110: 412-419, 2007.
34. Levi Z, Kark JD, Katz LH, Twig G, Derazne E, Tzur D, Leibovici Weissman Y, Leiba A, Lipshiez I, Keinan Boker L and Afek A: Adolescent body mass index and risk of colon and rectal cancer in a cohort of 1.79 million Israeli men and women: A population-based study. Cancer 123: 4022-4030, 2017.

35. Levi Z, Kark JD, Barchana M, Liphshitz I, Zavdy O, Tzur D, Derazne E, Furman M, Niv Y, Gordon B, Afek A and Shamiss A: Measured body mass index in adolescence and the incidence of colorectal cancer in a cohort of 1.1 million males. Cancer Epidemiol Biomarkers Prev 20: 2524-2531, 2011.
36. Chute CG, Willett WC, Colditz GA, Stampfer MJ, Baron JA, Rosner B and Speizer FE: A prospective study of body mass, height, and smoking on the risk of colorectal cancer in women. Cancer Causes Control 2: 117-124, 1991.
37. Hughes LA, Simons CC, van den Brandt PA, Goldbohm RA, de Goeij AF, de Bruine AP, van Engeland M and Weijenberg MP: Body size, physical activity and risk of colorectal cancer with or without the CpG island methylator phenotype (CIMP). PLoS One 6: e18571, 2011.
38. Jenniskens JCA, Offermans K, Simons C, Samarska I, Fazzi GE, van der Meer JRM, Smits KM, Schouten LJ, Weijenberg MP, Grabsch HI and van den Brandt PA: Energy balance-related factors in childhood and adolescence and risk of colorectal cancer based on KRAS, PIK3CA, and BRAF mutations and MMR status. Mol Carcinog 61: 1099-1115, 2022.
39. Jenniskens JCA, Offermans K, Simons C, Samarska I, Fazzi GE, Smits KM, Schouten LJ, Weijenberg MP, Grabsch HI and van den Brandt PA: Energy balance-related factors in childhood and adolescence and risk of colorectal cancer expressing different levels of proteins involved in the Warburg-effect. Int J Cancer 150: 1812-1824, 2022.
40. Simons CC, van den Brandt PA, Stehouwer CD, van Engeland M and Weijenberg MP: Body size, physical activity, early-life energy restriction, and associations with methylated insulin-like growth factor-binding protein genes in colorectal cancer. Cancer Epidemiol Biomarkers Prev 23: 1852-1862, 2014.
41. Gausman V, Liang PS, O'Connell K, Kantor ED and Du M: Evaluation of Early-Life Factors and Early-Onset Colorectal Cancer Among Men and Women in the UK Biobank. Gastroenterology 162: 981-983 e983, 2022.
42. Li H, Boakye D, Chen X, Jansen L, Chang-Claude J, Hoffmeister M and Brenner H: Associations of Body Mass Index at Different Ages With Early-Onset Colorectal Cancer. Gastroenterology 162: 1088-1097 e1083, 2022.
43. Chang VC, Cotterchio M, De P and Tinmouth J: Risk factors for early-onset colorectal cancer: a population-based case-control study in Ontario, Canada. Cancer Causes Control 32: 1063-1083, 2021.
44. Russo A, Franceschi S, La Vecchia C, Dal Maso L, Montella M, Conti E, Giacosa A, Falcini F and Negri E: Body size and colorectal-cancer risk. Int J Cancer 78: 161-165, 1998.
45. Boyle T, Fritschi L, Tabatabaei SM, Ringwald K and Heyworth JS: Smoking, alcohol, diabetes, obesity, socioeconomic status, and the risk of colorectal cancer in a population-based case-control study. Cancer Causes Control 25: 1659-1668, 2014.
46. Ishimaru S, Mimori K, Yamamoto K, Inoue H, Imoto S, Kawano S, Yamaguchi R, Sato T, Toh H, Iinuma H, Maeda T, Ishii H, Suzuki S, Tokudome S, Watanabe M, Tanaka J, Kudo SE, Sugihara K, Hase K, Mochizuki H, Kusunoki M, Yamada K, Shimada Y, Moriya Y, Barnard GF, Miyano S and Mori M: Increased risk for CRC in diabetic patients with the nonrisk allele of SNPs at 8q24. Ann Surg Oncol 19: 2853-2858, 2012.
47. Campbell PT, Jacobs ET, Ulrich CM, Figueiredo JC, Poynter JN, McLaughlin JR, Haile RW, Jacobs EJ, Newcomb PA, Potter JD, Le Marchand L, Green RC, Parfrey P, Younghusband HB, Cotterchio M, Gallinger S, Jenkins MA, Hopper JL, Baron JA, Thibodeau SN, Lindor NM, Limburg PJ, Martinez ME and Colon Cancer Family R: Case-control study of overweight, obesity, and colorectal cancer risk, overall and by tumor microsatellite instability status. J Natl Cancer Inst 102: 391-400, 2010.
48. Campbell PT, Cotterchio M, Dicks E, Parfrey P, Gallinger S and McLaughlin JR: Excess body weight and colorectal cancer risk in Canada: associations in subgroups of clinically defined familial risk of cancer. Cancer Epidemiol Biomarkers Prev 16: 1735-1744, 2007.

49. Chen C, Chen X, Wu D, Wang H, Wang C, Shen J, An Y, Zhong R, Li C and Liang W: Association of birth weight with cancer risk: a dose-response meta-analysis and Mendelian randomization study. J Cancer Res Clin Oncol 149: 3925-3935, 2023.

50. Cornish AJ, Law PJ, Timofeeva M, Palin K, Farrington SM, Palles C, Jenkins MA, Casey G, Brenner H, Chang-Claude J, Hoffmeister M, Kirac I, Maughan T, Brezina S, Gsur A, Cheadle JP, Aaltonen LA, Tomlinson I, Dunlop MG and Houlston RS: Modifiable pathways for colorectal cancer: a mendelian randomisation analysis. Lancet Gastroenterol Hepatol 5: 55-62, 2020.

51. Gao C, Patel CJ, Michailidou K, Peters U, Gong J, Schildkraut J, Schumacher FR, Zheng W, Boffetta P, Stucker I, Willett W, Gruber S, Easton DF, Hunter DJ, Sellers TA, Haiman C, Henderson BE, Hung RJ, Amos C, Pierce BL, Lindstrom S, Kraft P, the Colorectal Transdisciplinary S, Discovery B, Risk of Inherited Variants in Breast C, Elucidating Loci Involved in Prostate Cancer S, Follow-up of Ovarian Cancer Genetic A, Interaction S and Transdisciplinary Research in Cancer of the L: Mendelian randomization study of adiposity-related traits and risk of breast, ovarian, prostate, lung and colorectal cancer. Int J Epidemiol 45: 896-908, 2016.

52. Jarvis D, Mitchell JS, Law PJ, Palin K, Tuupanen S, Gylfe A, Hanninen UA, Cajuso T, Tanskanen T, Kondelin J, Kaasinen E, Sarin AP, Kaprio J, Eriksson JG, Rissanen H, Knekt P, Pukkala E, Jousilahti P, Salomaa V, Ripatti S, Palotie A, Jarvinen H, Renkonen-Sinisalo L, Lepisto A, Bohm J, Meklin JP, Al-Tassan NA, Palles C, Martin L, Barclay E, Farrington SM, Timofeeva MN, Meyer BF, Wakil SM, Campbell H, Smith CG, Idziaszczyk S, Maughan TS, Kaplan R, Kerr R, Kerr D, Buchanan DD, Win AK, Hopper JL, Jenkins MA, Lindor NM, Newcomb PA, Gallinger S, Conti D, Schumacher F, Casey G, Taipale J, Aaltonen LA, Cheadle JP, Dunlop MG, Tomlinson IP and Houlston RS: Mendelian randomisation analysis strongly implicates adiposity with risk of developing colorectal cancer. Br J Cancer 115: 266-272, 2016.

53. Fang X, Wang X, Song Z, Han D, Yin X, Liu B, Chen L, Zhang R, Lian F and Sui X: Causal association of childhood obesity with cancer risk in adulthood: A Mendelian randomization study. Int J Cancer 149: 1421-1425, 2021.

54. Papadimitriou N, Bull CJ, Jenab M, Hughes DJ, Bell JA, Sanderson E, Timpson NJ, Smith GD, Albanes D, Campbell PT, Kury S, Le Marchand L, Ulrich CM, Visvanathan K, Figueiredo JC, Newcomb PA, Pai RK, Peters U, Tsilidis KK, Boer JMA, Vincent EE, Mariosa D, Gunter MJ, Richardson TG and Murphy N: Separating the effects of early and later life adiposity on colorectal cancer risk: a Mendelian randomization study. BMC Med 21: 5, 2023.
